# The small molecule raptinal can simultaneously induce apoptosis and inhibit PANX1 activity

**DOI:** 10.1038/s41419-024-06513-z

**Published:** 2024-02-09

**Authors:** Jascinta P. Santavanond, Yu-Hsin Chiu, Rochelle Tixeira, Zonghan Liu, Jeremy K. Y. Yap, Kaiwen W. Chen, Chen-Lu Li, Yi-Ru Lu, Joan Roncero-Carol, Esteban Hoijman, Stephanie F. Rutter, Bo Shi, Gemma F. Ryan, Amy L. Hodge, Sarah Caruso, Amy A. Baxter, Dilara C. Ozkocak, Chad Johnson, Zoe I. Day, Alyce J. Mayfosh, Mark D. Hulett, Thanh K. Phan, Georgia K. Atkin-Smith, Ivan K. H. Poon

**Affiliations:** 1 Department of Biochemistry and Chemistry, La Trobe Institute for Molecular Science, La Trobe University, Melbourne, Victoria 3086 Australia; 2 Research Centre of Extracellular Vesicles, La Trobe University, Melbourne, Victoria Australia; 3 Departments of Medical Science, Life Science, and Medicine, National Tsing Hua University, Hsinchu, Taiwan; 4 Institute of Biotechnology, National Tsing Hua University, Hsinchu, Taiwan; 5 Unit for Cell Clearance in Health and Disease, VIB Center for Inflammation Research, Ghent, Belgium; 6 Department of Biomedical Molecular Biology, Ghent University, Ghent, Belgium; 7 Immunology Translational Research Programme, Department of Microbiology and Immunology, Yong Loo Lin School of Medicine, National University of Singapore, Singapore, Singapore; 8 Immunology Programme, Life Sciences Institute, National University of Singapore, Singapore, Singapore; 9 Regenerative Medicine Program, Bellvitge Institute for Biomedical Research (IDIBELL), Barcelona, Spain; 10 Department of Pathology and Experimental Therapeutics, Faculty of Medicine and Health Sciences, University of Barcelona, Barcelona, Spain; 11 The Walter and Eliza Hall Institute of Medial Research, Parkville, Vic Australia; 12 University of Melbourne, Melbourne, VIC Australia

**Keywords:** Apoptosis, Cell death

## Abstract

Discovery of new small molecules that can activate distinct programmed cell death pathway is of significant interest as a research tool and for the development of novel therapeutics for pathological conditions such as cancer and infectious diseases. The small molecule raptinal was discovered as a pro-apoptotic compound that can rapidly trigger apoptosis by promoting the release of cytochrome c from the mitochondria and subsequently activating the intrinsic apoptotic pathway. As raptinal is very effective at inducing apoptosis in a variety of different cell types in vitro and in vivo, it has been used in many studies investigating cell death as well as the clearance of dying cells. While examining raptinal as an apoptosis inducer, we unexpectedly identified that in addition to its pro-apoptotic activities, raptinal can also inhibit the activity of caspase-activated Pannexin 1 (PANX1), a ubiquitously expressed transmembrane channel that regulates many cell death-associated processes. By implementing numerous biochemical, cell biological and electrophysiological approaches, we discovered that raptinal can simultaneously induce apoptosis and inhibit PANX1 activity. Surprisingly, raptinal was found to inhibit cleavage-activated PANX1 via a mechanism distinct to other well-described PANX1 inhibitors such as carbenoxolone and trovafloxacin. Furthermore, raptinal also interfered with PANX1-regulated apoptotic processes including the release of the ‘find-me’ signal ATP, the formation of apoptotic cell-derived extracellular vesicles, as well as NLRP3 inflammasome activation. Taken together, these data identify raptinal as the first compound that can simultaneously induce apoptosis and inhibit PANX1 channels. This has broad implications for the use of raptinal in cell death studies as well as in the development new PANX1 inhibitors.

## Introduction

With the rapid expansion of the cell death field, apoptosis remains the most characterised form of programmed cell death [1, 2]. Apoptosis is regulated by one of two pathways: the extrinsic death receptor pathway or the intrinsic mitochondrial pathway [3]. The extrinsic pathway requires the binding of ligands to transmembrane death receptors, subsequently initiating a series of downstream cascade events. In contrast, the intrinsic pathway is triggered by various non-receptor-mediated stimuli that cause DNA damage, reactive oxygen species production or endoplasmic reticulum stress. This leads to mitochondrial outer membrane permeabilisation driven by pro-apoptotic BCL-2 proteins such as BAX, BAK and BOK, resulting in the release of cytosolic apoptogenic factors including cytochrome c [1, 3]. These two distinct pathways ultimately converge to activate caspases 3 and 7 and orchestrate the executioner phase of apoptosis [3, 4].

As too much or too little cell death is implicated in a number of disease settings [3], the identification of novel apoptosis inducing agents are of significant interest to the cell death field for the study of apoptotic machinery and development of novel therapeutics. In conditions such as cancer where there is inadequate cell death, chemotherapy drugs including venetoclax (BH3 mimetic) [5, 6], doxorubicin (topoisomerase inhibitor) and cisplatin (DNA cross-linker) are commonly used to induce apoptosis of malignant cells [7]. Whilst these drugs are extremely effective against a range of cancers, the clinical usage is limited due to potential side effects such as renal and cardiac toxicities [7–9]. Additionally, the emergence of multidrug resistance highlights the necessity of identifying novel apoptosis inducing drugs to kill resistant tumour cells [10, 11]. Raptinal, a small lipophilic molecule, was discovered in 2015 as a compound that can induce intrinsic apoptosis within minutes in a wide range of cell lines by directly disrupting mitochondrial function and promoting the release of cytochrome c, independent of pro-apoptotic factors such as BAX, BAK and BOK [12, 13]. Thus, raptinal can trigger the intrinsic apoptosis pathway rapidly in cells through bypassing upstream signalling events. Since its discovery, raptinal has been used in a wide range of in vitro studies investigating various cell death modalities such as apoptosis [14, 15], ferroptosis [16], pyroptosis [17–19] as well as apoptotic cell clearance [20]. Furthermore, raptinal has also been demonstrated as a potent apoptosis inducer in model organisms such as zebrafish [12, 20] and a pre-clinical mouse model of hepatocellular carcinoma [21].

In addition to the pro-apoptotic properties of raptinal, here we discovered that raptinal is a novel inhibitor of the plasma membrane channel Pannexin 1 (PANX1). PANX1 is a ubiquitously expressed heptameric plasma membrane channel that can facilitate the release of metabolites such as ATP from both healthy and dying cells in a variety of physiological and pathological settings [22, 23]. In the context of cell death and clearance, PANX1 channels can be activated by caspase 3/7-mediated cleavage and facilitate the release of chemotactic ‘find-me’ signals (metabolites such as ATP and UTP) to aid the recruitment of phagocytes to the site of cell death [24]. Caspase-activated PANX1 also aids the release of ‘good-bye’ signals (metabolites such as spermidine) to establish an anti-inflammatory environment and promote wound healing responses [25]. Furthermore, PANX1 activation regulates the fragmentation of dying cells [26] and inflammasome activation [27, 28]. By monitoring the activity and function of caspase-activated PANX1 channels, we found that raptinal not only induces apoptosis but can also concurrently block PANX1-dependent release of ‘find-me’ signals and selective uptake of the cell-impermeable dye TO-PRO-3 by apoptotic cells. Raptinal treatment can also interfere with PANX1 regulated apoptotic cell disassembly and inflammasome activation during apoptosis. The discovery of raptinal as a new inhibitor of PANX1 has broad implications for its use in cell death studies and represents the first known compound that can simultaneously induce apoptosis and inhibit PANX1 channels.

## Results

### Absence of TO-PRO-3 uptake and ATP release through caspase-activated PANX1 in cells treated with raptinal to induce apoptosis

**Fig. 1:**
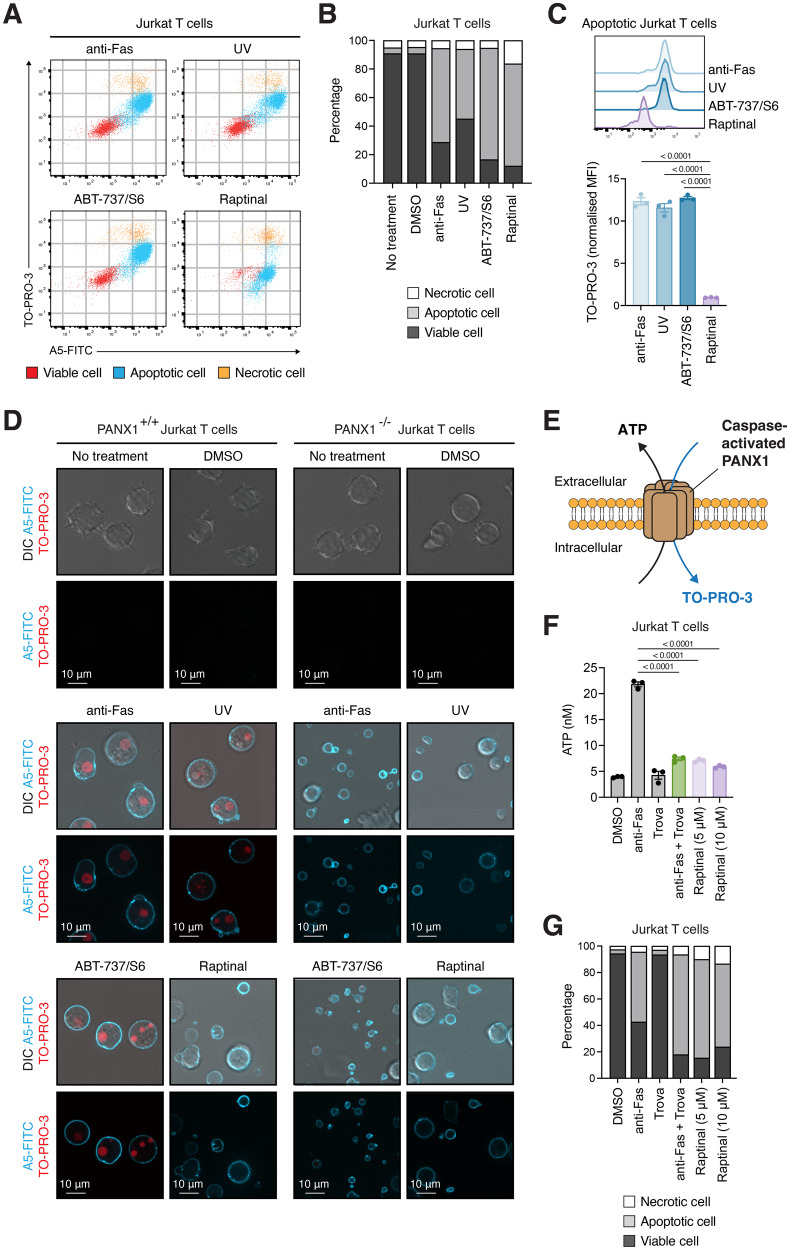
Lack of TO-PRO-3 uptake and ATP release by cells treated with raptinal to induce apoptosis. **A** Flow cytometry analysis of Jurkat T cells treated with different apoptotic stimuli including anti-Fas (250 ng/mL), UV irradiation (150 mJ cm^-2^), ABT-737 (5 μM)/S63845 (0.5 μM) or raptinal (10 μM) for 4 h. Apoptosis markers such as the exposure of phosphatidylserine on the cell surface and PANX1 channel activation was monitored by A5 and TO-PRO-3 staining, respectively. **B** Quantification of the relative levels of viable, apoptotic and necrotic cells. (*n* = 3) **C** Flow cytometry analysis of TO-PRO-3 uptake by apoptotic Jurkat T cells treated with different apoptotic stimuli as per (A). (*n* = 3) Error bars represent s.e.m. **D** Confocal microscopy images monitoring PANX1^+/+^ and PANX1^−/−^ Jurkat T cells treated with different apoptotic stimuli as per for 4 h (A). Apoptosis markers were assessed as per (A). (*n* = 3) **E** Schematic of caspase-activated PANX1 mediating the release of ATP and uptake of TO-PRO-3 during apoptosis. **F** ATP release from Jurkat T cells treated with anti-Fas (250 ng/mL), trova (20 μM, PANX1 inhibitor), anti-Fas (250 ng/mL) and trova (20 μM) or raptinal (5 or 10 μM) for 4 h. (*n* = 3) Error bars represent s.e.m. **G** Flow cytometry analysis of Jurkat T cells treated with anti-Fas (250 ng/mL) in presence or absence of trova (20 μM) or raptinal (5 or 10 μM) for 4 h to determine the relative levels of viable, apoptotic and necrotic cells. (*n* = 3) Data are representative of three independent experiments. One-way ANOVA followed by Dunnett test was performed to determine the indicated *p-*value.

Raptinal is a small molecule that can rapidly induce apoptosis in vitro and in vivo [12]. To confirm the pro-apoptotic properties of raptinal, we treated human Jurkat T cells with raptinal or other apoptosis inducers such as anti-Fas, UV irradiation or a combination of the BH3 mimetic ABT-737 and the MCL-1 inhibitor S63845 (ABT-737/S6). Cells at different stages of cell death were identified based on our previously established flow cytometry approach, in which apoptotic cells are able to bind annexin A5 (A5), indicative of phosphatidylserine (PtdSer) exposure, and uptake an intermediate level of the membrane impermeable DNA-binding dye TO-PRO-3 through caspase-activated PANX1 membrane channels [29]. Induction of extrinsic (anti-Fas treatment) or intrinsic (UV irradiation, ABT-737/S6 and raptinal treatment) apoptosis in Jurkat T cells led to a marked increase in apoptotic cells (Fig. 1A and B, Supplementary Fig. 1). Surprisingly, we observed that TO-PRO-3 uptake by apoptotic cells was significantly reduced for raptinal treated cells (Fig. 1A and C), despite comparable levels of viable, apoptotic, and necrotic cells across each apoptotic stimulus (Fig. 1B). Reduced TO-PRO-3 uptake by apoptotic cells and fragments following raptinal but not anti-Fas, UV, ABT-737/S6 treatment were also confirmed by confocal microscopy analysis (Fig. 1D). Notably, the absence of TO-PRO-3 uptake by apoptotic cells and fragments treated with raptinal resembled apoptotic PANX1 deficient (PANX1^−/−^) Jurkat T cells treated with various apoptotic stimuli (Fig. 1D), suggesting that raptinal may interfere with the activity of caspase-activated PANX1 channels. Reduction of TO-PRO-3 uptake by apoptotic cells was also observed for primary mouse thymocytes and zebrafish embryonic cells treated with raptinal (Supplementary Fig. 2A-C). Furthermore, activation of PANX1 can facilitate the release of metabolites such as ATP from apoptotic cells (Fig. 1E), which can function as a ‘find-me’ signal to aid the recruitment of phagocytes towards apoptotic cells during cell clearance [29]. Under apoptotic conditions, cells treated with raptinal or the previously described PANX1 inhibitor trovafloxacin (trova) [26] also showed reduced levels of ATP in the cultured supernatant (Fig. 1F, G). Collectively, these data demonstrate that raptinal not only is a potent inducer of apoptosis but apoptotic cells treated with raptinal also displayed a lack of PANX1 activities.

Caspase-mediated processing of PANX1 is required for its activation during apoptosis [29]. Proteolytic cleavage of full-length PANX1 (~45–50 kDa) to a distinct N-terminus containing caspase-cleaved fragment (~15 kDa) was observed in Jurkat T cells treated with raptinal or anti-Fas to induce apoptosis, as determined by immunoblot analysis (Supplementary Fig. 3A). Next, we generated a Jurkat T cell line expressing PANX1 fused with GFP at the C-terminus (PANX1-GFP, Supplementary Fig. 3B and 3C) to monitor the processing of PANX1 C-terminal caspase-cleavage site during apoptosis by time-lapse confocal microscopy. PANX1-GFP showed localisation at the plasma membrane prior to apoptosis induction and a diffused GFP signal throughout the cell during UV-induced apoptosis as indicated by dynamic membrane blebbing (Supplementary Fig. 3D). Similarly, Jurkat T cells expressing PANX1-GFP treated with raptinal or anti-Fas also resulted in diffused localisation of GFP during apoptosis (Supplementary Fig. 3E). Notably, apoptosis induction by raptinal or anti-Fas, as well as the change in GFP localisation were both prevented by treatment with pan-caspase inhibitor Q-VD-OPh (Supplementary Fig. 3E). Thus, these data suggest that caspase-mediated PANX1 cleavage can occur effectively during raptinal-induced apoptosis and the effects of raptinal treatment on TO-PRO-3 uptake and ATP release by apoptotic cells as described above is not due to the lack of caspase-mediated processing of PANX1. Notably, as the level of caspase-cleaved N-terminal fragment of PANX1 were comparable between raptinal-treated cells and other apoptotic conditions (Supplementary Fig. 3A), we also reasoned that raptinal treatment is unlikely to alter PANX1 expression.

### Raptinal exhibits unique properties in blocking caspase-activated PANX1 in apoptotic cells

**Fig. 2:**
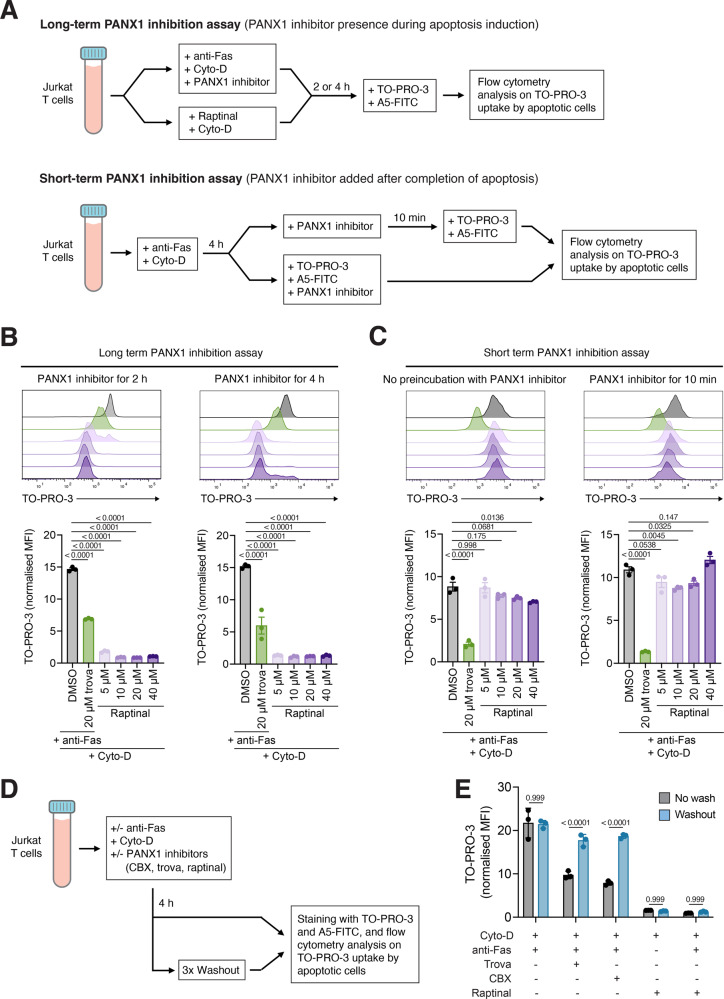
Raptinal exhibit unique properties in blocking caspase-activated PANX1 in apoptotic cells. **A** Schematic detailing the experimental approaches used to determine whether PANX1 channels on apoptotic cells are blocked following long- or short-term treatment with PANX1 inhibitors. Jurkat T cells were treated with anti-Fas (250 ng/mL) or the indicated concentration of raptinal to induce apoptosis (2 or 4 h). Cells were also treated with Cyto-D (10 μM) to prevent apoptotic cell fragmentation. Cells were stained with A5-FITC and TO-PRO-3 to monitor apoptosis induction and PANX1 activity, respectively, as determined by flow cytometry analysis. **B**, **C** Flow cytometry analysis of TO-PRO-3 uptake by apoptotic cells following **B** long-term treatment or **C** short-term treatment with the indicated concentration of raptinal or trova (20 μM). (*n* = 3) **D** Schematic detailing the washout experiment to determine whether PANX1 inhibitors can be washed out from apoptotic cells and restore TO-PRO-3 uptake. **E** Flow cytometry analysis of TO-PRO-3 uptake by apoptotic Jurkat T cells treated with various PANX1 inhibitors including trova (20 μM), CBX (50 μM) and raptinal (10 μM) with or without washing steps. Apoptosis was induced by anti-Fas (250 ng/mL) or raptinal (10 μM) for 4 h. (*n* = 3). **B**, **C**, **E** Error bars represent s.e.m. Data are representative of three independent experiments. Cyto-D (10 μM) was added in all experiments. One-way ANOVA followed by Dunnett test was performed for (**B**, **C**) and two-way ANOVA followed by Šidák test was performed for (**E**) to determine the indicated *p-*value.

To examine whether raptinal is a novel PANX1 inhibitor, we established two new experimental protocols to assess the impact of long- and short-term drug treatments on PANX1 activities based on TO-PRO-3 uptake by apoptotic cells. Briefly, Jurkat T cells were induced to undergo apoptosis with PANX1 inhibitors present at the time of apoptosis induction (i.e. long-term PANX1 inhibition assay) or PANX1 inhibitors added at 4 h post apoptosis induction (i.e. short-term PANX1 inhibition assay) (Fig. 2A). For the short-term PANX1 inhibition assay, PANX1 inhibitors were added either 10 minutes prior to TO-PRO-3 staining or simultaneously with TO-PRO-3 staining. It should be noted that since PANX1 inhibitors can also promote apoptotic cell fragmentation [26], all samples were treated with cytochalasin D (Cyto-D) to prevent the cell fragmentation process to ensure accurate quantification of TO-PRO-3 uptake by apoptotic cells (Fig. 2A). In both long- and short-term PANX1 inhibition assays, trova treatment significantly blocked TO-PRO-3 uptake by apoptotic cells (Fig. 2B, C). Interestingly, although raptinal treatment was able to significantly inhibit TO-PRO-3 uptake in the long-term PANX1 inhibition assay (to an even greater extent than trova treatment) (Fig. 2B), raptinal was not effective in reducing TO-PRO-3 uptake in the short-term PANX1 inhibition assay (Fig. 2C). Next, we examined whether PANX1 inhibitors such as trova, carbenoxolone (CBX) and raptinal can be washed out from apoptotic cells and restore TO-PRO-3 uptake (Fig. 2D). Following three washout steps, TO-PRO-3 uptake by apoptotic cells treated with trova or CBX was restored to a comparable level as apoptotic cells without PANX1 inhibitor treatment (Fig. 2E). In contrast, the washout steps did not restore TO-PRO-3 uptake by apoptotic cells treated with raptinal (Fig. 2E). Collectively, these data suggest that raptinal may inhibit caspase-activated PANX1 channels via a distinct mechanism to other known PANX1 inhibitors such as trova and CBX.

**Fig. 3:**
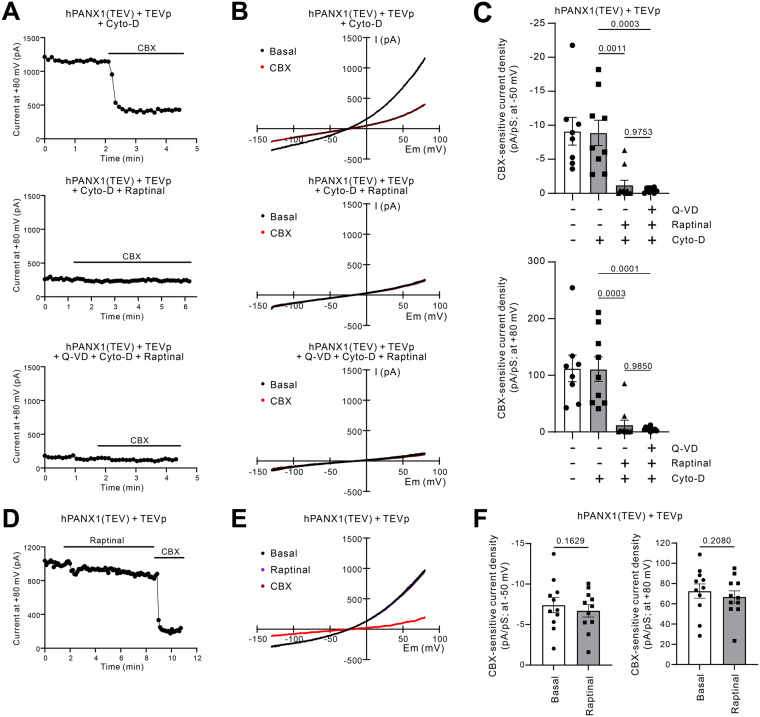
Raptinal inhibits cleavage-mediated plasma membrane PANX1 outward and inward currents. **A** Time series of whole-cell currents from HEK293T cells expressing hPANX1(TEV) and TEVp at +80 mV with or without raptinal (10 μM) pre-treatment (middle and top panels, respectively), or in the presence of Q-VD-OPh (10 μM) pre-incubation 1 h prior to raptinal treatment (bottom panel). Cells were treated with the PANX1 inhibitor CBX (50 μM) to determine the CBX-sensitive current density. Cells were also treated with Cyto-D (10 μM) to prevent fragmentation. **B** Current-voltage relationships (I-V curves) from HEK293T cells expressing hPANX1(TEV) and TEVp with or without raptinal (10 μM) pre-treatment (middle and top panels, respectively), or in the presence of Q-VD-OPh (10 μM) pre-incubation 1 h prior to raptinal treatment (bottom panel). **C** Quantification of CBX-sensitive current density at -50 mV (top) and +80 mV (bottom) with or without raptinal or Q-VD-OPh pre-treatment. Error bars represent s.e.m. Numbers indicate *p-*values derived from one-way ANOVA with Tukey’s multiple comparison test. **D** Time series of whole-cell currents from HEK293T cells expressing hPANX1(TEV) and TEVp at +80 mV treated with raptinal (10 μM) and subsequently CBX (50 μM) by bath application. **E** Current-voltage relationships from experiment described in (**D**). **F** Quantification of CBX-sensitive current density at +80 mV and -50 mV treated with raptinal by bath application. Unpaired student’s two tailed *t*-test was performed to determine the indicated *p*-values. Error bars represent s.e.m. Data are grouped from >3 independent experiments.

To further investigate the inhibition of PANX1 by raptinal, we measured cleavage-mediated plasma membrane PANX1 outward current (at +80 mV) and inward current (at -50 mV) by whole-cell patch-clamp recordings in a previously described HEK293T cells system [30]. Briefly, membrane current of PANX1 was measured in HEK293T cells co-expressing human PANX1 with the C-terminal caspase cleavage site substituted with a tobacco etch virus (TEV) protease cleavage site (denoted as hPANX1(TEV)) and TEV protease (TEVp) [30]. Similar to the long-term PANX1 inhibition assay as described above, when cells were pre-treated with raptinal for 1 h prior to channel recording, both the outward and inward membrane currents of cleaved-PANX1 were abolished (Fig. 3A–C). Additionally, pre-treating cells with pan-caspase inhibitor Q-VD-OPh to prevent apoptosis induction and caspase-mediated cleavage of PANX1 by raptinal (Supplementary Fig. 4), also led to the inhibition of outward and inward membrane currents (Fig. 3A–C). In contrast, when cells were treated with raptinal whilst PANX1 currents were being recorded (analogous the short-term PANX1 inhibition assay), raptinal had no effect on the outward and inward currents (Fig. 3D–F). It should be noted that the well-described PANX1 inhibitor CBX blocked both the outward and inward currents under all conditions tested (Fig. 3A, B, D and E). Collectively, these data further demonstrate that raptinal can inhibit the activity of cleavage-activated PANX1 channels but the mode of action could be distinct to other PANX1 inhibitors. Furthermore, the inhibitory effects of raptinal is unlikely to be associated with the pro-apoptotic properties of raptinal.

### Raptinal promotes apoptotic body formation during apoptosis induction

**Fig. 4:**
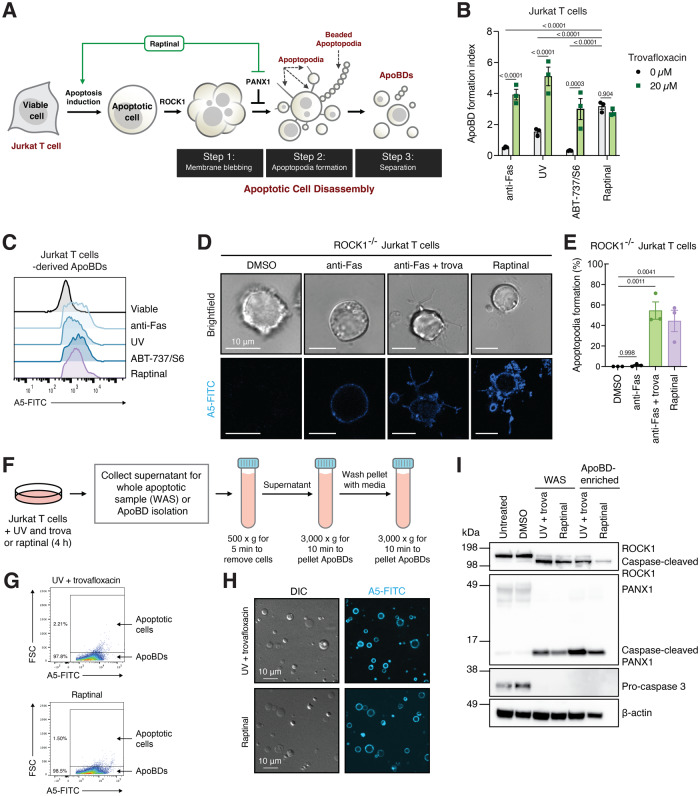
Raptinal promotes ApoBD formation during apoptosis induction. **A** Schematic of how raptinal could impact the apoptotic cell disassembly process by inducing apoptosis and blocking PANX1 function. **B** Formation of ApoBD by Jurkat T cells treated with various apoptotic stimuli (anti-Fas (250 ng/mL), UV irradiation (150 mJ cm^-2^), ABT-737 (5 μM)/S63845 (0.5 μM) or raptinal (10 μM) for 4 h) in the presence or absence of trova (20 μM), as measured by flow cytometry analysis. (*n* = 3) Error bars represent s.e.m. **C** Relative PtdSer exposure levels on ApoBDs derived from Jurkat T cells treated with various apoptotic stimuli (as per (B)), as determined by A5-FITC staining. **D** Confocal microscopy images monitoring ROCK1^−/−^ Jurkat T cells treated with anti-Fas (250 ng/mL), anti-Fas (250 ng/mL) and trova (20 μM), or raptinal (10 μM) for 4 h. White arrow heads indicate apoptopodia formation. **E** Quantitation of live microscopy data from (**D**) to determine the proportion of cells able to generate apoptopodia during apoptosis. **F** Schematic of the isolation of ApoBD by differential centrifugation. **G**, **H** ApoBDs generated from Jurkat T cells treated with UV (150 mJ cm^-2^) and trova (20 μM) or raptinal (10 μM) for 4 h were enriched by differential centrifugation and ApoBD purity determined by flow cytometry and ApoBD quality by confocal microscopy. **I** Immunoblot analysis of whole apoptotic sample and ApoBD-enriched samples as described in (**F**). Jurkat T cells were induced to undergo apoptosis as per (**G**). **C**, **D**, **G**, **H**, **I** Data are representative of three independent experiments. Two-way ANOVA followed by Šidák test was performed for (**B**) and one-way ANOVA followed by Dunnett test was performed for (**E**) to determine the indicated *p-*value.

During apoptosis, dying cells undergo cell fragmentation to generate a type of large extracellular vesicles (EVs) known as apoptotic bodies (ApoBDs) [31]. The formation of ApoBDs is regulated by distinct morphological steps including membrane blebbing, apoptopodia formation and separation into distinct ApoBDs [32] (Fig. 4A). Notably, the generation of apoptopodia, a thin string-like membrane protrusion, and subsequently ApoBD formation is negatively regulated by caspase-activated PANX1 channels [26]. Therefore, we examined whether raptinal could induce apoptosis and concurrently promote ApoBD formation through PANX1 inhibition (Fig. 4A). In comparison to other apoptotic stimuli, raptinal treatment generated the highest level of ApoBD formation, as determined by flow cytometry analysis [33] (Fig. 4B). Furthermore, treatment with the PANX1 inhibitor trova promoted ApoBD formation when cells were induced to undergo apoptosis by anti-Fas, UV or ABT-737/S6 but not raptinal (Fig. 4B), suggesting that PANX1 channels are already blocked when cells were treated with raptinal. It should be noted that ApoBDs generated under all apoptotic conditions exposed comparable level of PtdSer as determined by A5 staining (Fig. 4C). Notably, confocal microscopy analysis of Jurkat T cells treated with various apoptotic stimuli also demonstrated that raptinal treated cells underwent more extensive apoptotic cell disassembly as compared to anti-Fas, UV or ABT-737/S6 treated cells (Fig. 1D, Supplementary Fig. 5). To directly investigate whether PANX1 blockade by raptinal could promote the formation of apoptopodia, time-lapse confocal microscopy was performed using the ROCK1^−/−^ Jurkat T cells to enable clear visualisation of apoptopodia without dynamic membrane blebbing as described previously [26, 34]. ROCK1^−/−^ Jurkat T cells treated with raptinal or anti-Fas in the presence of trova frequently generated apoptopodia during the progression of apoptosis, whereas cells treated with anti-Fas only rarely formed apoptopodia (Fig. 4D, E, Supplementary Fig. 6). Next, we characterised ApoBDs generated by raptinal treated cells based on previously established criteria for apoptotic EV characterisation [35]. ApoBDs were first isolated by differential centrifugation (Fig. 4F), as described previously [36, 37]. Similar to ApoBDs generated from Jurkat T cells treated with UV and trova, ApoBDs derived from cells treated with raptinal were isolated to high purity (>97%) and exhibited PtdSer exposure (Fig. 4G, H). Caspase-cleaved proteins such as ROCK1 and PANX1 were also present in ApoBDs as determined by immunoblot analysis (Fig. 4I). Taken together, these data suggest that raptinal can concurrently induce apoptosis and enhance the formation of large apoptotic EVs such as ApoBDs.

### Raptinal inhibits inflammasome activation during apoptosis induction

**Fig. 5:**
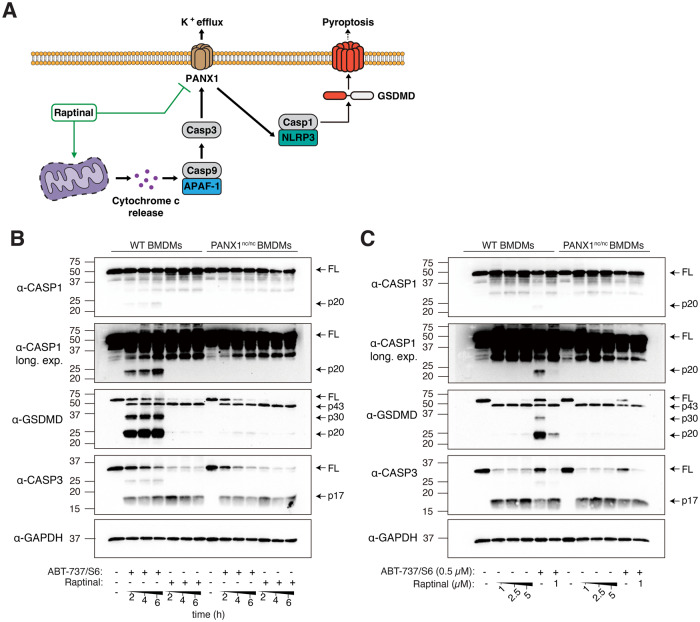
Raptinal inhibits inflammasome activation during apoptosis induction. **A** Schematic of how raptinal could impact NLRP3 inflammasome, Casp-1 and GSDMD activation by inducing apoptosis and blocking PANX1 function. **B** BMDMs from WT and PANX1^nc/nc^ (PANX1 non-cleavable) mice were treated with ABT-737/S6 (500 nM) or raptinal (10 μM) over 6 h, and mixed supernatant and extracts were analysed by immunoblot. **C** BMDMs from WT and PANX1^nc/nc^ mice were treated with the indicated concentration of ABT-737/S6 and/or raptinal for 6 h, and mixed supernatant and extracts were analysed by immunoblot. Data are representative of three independent experiments.

In addition to regulating the release of ‘find-me’ signals (Fig. 1E) and ApoBD formation (Fig. 4A), activation of PANX1 channels during apoptosis has also been shown to promote NLRP3 activation and subsequent processing of caspase 1 and gasdermin-D (GSDMD) to drive downstream inflammatory processes [28] (Fig. 5A). To investigate whether raptinal can concurrently induce intrinsic apoptosis and limit the activation of NLRP3, we treated bone marrow-derived macrophages (BMDMs) with raptinal and assessed the level of cell death and caspase 1 and GSDMD processing, respectively. BMDMs from WT mice treated with raptinal or ABT-737/S6 to induce mitochondrial apoptosis exhibited comparable levels of cell death at 6 h post treatment as indicated by LDH release (Supplementary Fig. 7) and caspase 3 activation (Fig. 5B). However, unlike ABT-737/S6 treatment, raptinal-treated WT BMDMs did not promote the processing of caspase 1 or GSDMD (Fig. 5B). Notably, processing of caspase 1 and GSDMD was also reduced in BMDMs from PANX1^nc/nc^ mice (C-terminal caspase-cleavage site of PANX1 is mutated and is resistant to caspase cleavage [38]) treated with raptinal or ABT-737/S6 (Fig. 5B), further demonstrating the importance of caspase-activated PANX1 in regulating NLRP3 activation following intrinsic apoptosis induction. Furthermore, BMDMs treated with both ABT-737/S6 and raptinal also showed reduced caspase 1 processing despite apoptosis being triggered effectively (Fig. 5C, Supplementary Fig. 7). Collectively, these data indicate that raptinal can induce intrinsic apoptosis without the activation of NLRP3 though PANX1 blockade.

## Discussion

Small molecules that can trigger apoptosis are widely used in research and in clinical settings, as exemplified by the BCL-2-specific BH3 mimetic venetoclax that is currently used to treat patients with acute myeloid leukaemia or chronic lymphocytic leukaemia [39]. As raptinal is very effective in inducing apoptosis in a variety of cell types in vitro and in vivo [12, 13], raptinal has been adopted across a diverse range of studies in the cell death and cell biology fields. This includes studies investigating cell death pathways including apoptosis [14, 15], pyroptosis [17–19], and ferroptosis [16] as well as in studies of dying cell clearance (i.e. efferocytosis) [20], autophagy [40], inflammasome activation [41], intercellular communication [42], extracellular matrix formation [43], anti-microbial compounds [44], and methodologies to monitor cell death progression [45]. Furthermore, studies have also investigated the use of raptinal as an anticancer agent in preclinical studies [46]. Therefore, the data presented in this study demonstrating that raptinal not only induces apoptosis but also simultaneously blocks a spectrum of PANX1-driven functions has significant implications on both previous studies and future use of raptinal. For example, PANX1 facilitates the release of ‘find-me’ signals to aid the recruitment of phagocytes towards the site of cell death [24, 29] (Fig. 1). PANX1 also mediates the release of ‘good-bye’ signals such as guanosine 5’-monophosphate and spermidine from apoptotic cells to alter the gene programmes of neighbouring immune cells to establish wound healing and anti-inflammatory responses [25]. During apoptosis, PANX1 negatively regulates the formation of ApoBDs [26] (Fig. 4) and can subsequently influence the efficiency of efferocytosis [34]. Caspase-activated PANX1 has also been shown to regulate the activation of noncanonical inflammasome and pyroptosis induction [47], and NLRP3 inflammasome activation during apoptosis [28] (Fig. 5). Furthermore, PANX1 was also found to regulate erastin-induced ferroptosis [48], autophagy [49] as well as the release of pro-inflammatory cytokines during necroptosis [50]. Thus, the ability of raptinal to inhibit PANX1 during cell death induction should be taken into consideration in various studies.

Through an unbiased drug screening approach, we have previously identified the antibiotic trova and the anti-hypertensive spironolactone as new inhibitors of PANX1 [26, 51]. Discovery of such properties of trova and spironolactone not only highlights the potential side effects of these drugs but also the opportunities to repurpose them to inhibit PANX1 in certain settings. Thus, besides considering how raptinal could impact the interpretation of results in previous studies and the need to cautiously use raptinal as an apoptosis inducer as noted above, the potential benefit of a small molecule that can induce both apoptosis and PANX1 blockade is an exciting possibility. For example, EVs generated from apoptotic cells have been shown to exhibit a variety of therapeutic properties including the ability to promote wound healing responses [52], and resolution of inflammation [53]. Thus, raptinal can be used as a single agent to induce apoptosis and promote the formation of ApoBDs through PANX1 blockade, which could be useful to generate a large amount of ApoBDs for therapeutic applications. Another interesting concept to consider is the consequence of inducing cancer cell death whilst modulating their removal by phagocytes, and/or metabolite release, and/or inflammasome activation through PANX1 blockade. Therefore, harnessing this dual function of raptinal could be beneficial in certain therapeutic settings and should be explored in future studies.

A number of structurally distinct PANX1 inhibitors have been described [54]. Relevant to the data presented in this study, inhibitors such as CBX and spironolactone can inhibit both inward and outward currents of PANX1 in patch-clamp assays [51]. In contrast, trova only inhibited the inward but not outward currents of PANX1 [26], although the underpinning mechanism is not well understood. Mechanistically, drugs such as CBX can bind directly to PANX1 via the aromatic side chain of W74 in the heptameric assembly of PANX1 and block the pore of the channel by occupying the entrance at the extracellular side of PANX1 [55]. Interestingly, TO-PRO-3 uptake and channel recording data presented in this study suggest raptinal could inhibit PANX1 via a distinct mechanism compared to trova and CBX. Particularly, how raptinal cannot be ‘washed off’ apoptotic cells, and the ability of raptinal to inhibit PANX1 only after pre-treatment but not acutely is intriguing. Whether these observations could be attributed to the electrophilic properties of raptinal and thus enabling it to covalently link to its target(s) in cells remains to be defined. Furthermore, due to the peculiar effects of raptinal on PANX1 activity as described in this study, it is unclear whether raptinal is acting directly or indirectly on PANX1 (e.g. via a metabolite of raptinal). Nevertheless, it is worth noting that the ability of raptinal to inhibit PANX1 is unlikely to be associated with its pro-apoptotic activities as blockade of apoptosis did not affect raptinal-mediated PANX1 inhibition, and targeting PANX1 pharmacologically or genetically did not accelerate apoptosis induction by other apoptotic stimulus. Although how raptinal can inhibit the activity of caspase-activated PANX1 is unclear and warrants further investigation, designing PANX1 inhibitors based on raptinal, in particular without the apoptosis inducing property could be an approach to identify new small molecules targeting PANX1 via a mechanism that is distinct to other well described PANX1 inhibitors such as CBX. Notably, whether raptinal can inhibit PANX1 activity in non-apoptotic settings should also be examined in future studies as PANX1 can also be activated via other mechanisms such as mechanical stress, increase in extracellular potassium as well as receptor-dependent signalling [56]. Since PANX1 has been implicated to play a role in many pathological conditions including cardiovascular, inflammatory, and neurological diseases [23], identifying new PANX1 inhibitors may lead to the development of novel therapeutics targeting these PANX1-associated diseases.

## Materials and Methods

### Reagents

Raptinal, anti-Fas (clone CH11), cytochalasin D (Cyto-D), carbenoxolone (CBX), trovafloxacin (trova), dimethyl sulfoxide (DMSO) and poly-l-lysine were purchased from Sigma-Aldrich. A5-FITC, A5-PE and A5 binding buffer were purchased from BD Bioscience. TO-PRO-3, RPMI 1640 medium, DMEM (1 g/L glucose) medium, Penicillin Streptomycin solution and Hoechst 33342 were purchased from Thermo-Fisher Scientific. Fetal calf serum (FCS) was purchased from Scientifix. ABT-737 and S63845 were purchased from Jomar Life Research.

### Cell culture

Human Jurkat T cells (ATCC), PANX1^−/−^ and ROCK1^−/−^ Jurkat T [34] were cultured in RPMI 1640 supplemented with 10% (v/v) foetal calf serum, 50 IU/mL penicillin and 50 μg/mL streptomycin. PANX1-GFP Jurkat T cells were generated by electroporation as described previously [29]. HEK293T cells (ATCC, P4 ~ P20) were cultured in DMEM (Gibco) containing 10% FBS (HyClone). For whole-cell recording, HEK293T were transiently co-transfected with plasmids encoding hPanx1(TEV)-EGFP and TEV protease using Lipofectamine 3000 following manufacturers’ manual. Cells were replated onto poly-L-lysine-coated coverslips 16-18 h after transfection. Bone marrow-derived macrophages were differentiated using L929 supernatant as previously described [28] and used between Day 7–9 of differentiation. BMDMs were seeded in 96-well plates at 5 × 10^4^ cells per well in complete DMEM a day prior to stimulation in Opti-MEM. All cell lines were incubated at 37°C in 5% CO_2_.

### Induction of apoptosis

To induce apoptosis by UV irradiation, Jurkat T cells were exposed to 150 mJ/cm^2^ of irradiation using the Stratagene UV Stratalinker 1800 (Agilent Technologies) and incubated for 4 h. For drug induced apoptosis, Jurkat T cells were treated with anti-Fas (250 ng/mL), or the indicated concentrations of raptinal, or BH3 mimetics ABT-737 and S63845 (ABT-737/S6) diluted in serum free RPMI or DMEM medium supplemented with 1% BSA and incubated for 4 h.

### Ex vivo analysis of TO-PRO-3 uptake by primary mouse thymocytes

C57BL/6 mice (male and female) were maintained and used under approval of AEC15–36, La Trobe University. All methods and experiments were approved by the La Trobe University Animal Ethics Committee in accordance with the National Health and Medical Research Council Australia code of practice for the care and use of animals for scientific purposes. Mice were euthanised by CO_2_ asphyxiation and the thymus was harvested and homogenised through a 70 μm cell strainer in 1% BSA/RPMI media to isolate thymocytes. Thymocytes were treated with or without raptinal (10 μM) or ABT-737/S6 for 4 h and analysed by flow cytometry (as described below).

### Ex vivo analysis of TO-PRO-3 uptake by dying zebrafish embryonic cells

Zebrafish were maintained and bred according to the standard procedures at the aquatic facility of the Bellvitge Biomedical Research Institute (IDIBELL). Embryos were kept in E3 medium at 28°C prior to experiments and staged based on morphological criteria. The protocol used has been approved by the Ethical Committee of Animal Experimentation of IDIBELL (CEEA IDIBELL) and was implemented according to national and European regulations. Females were crossed with males (between 5 and 12 months of age) to obtain eggs. Cultures of zebrafish embryonic cells were prepared at 4 hpf by vortexing manually dechorionated embryos, centrifugation, collection of the pellet in DMEM medium (DMEM/F-12 with L-Glutamine and HEPES, Sigma), and plating of the cells for live imaging on a glass bottom dish (MatTek) in presence of 0.5 µM TO-PRO-3. Cells were obtained from AB wild-type embryos co-injected with 50 pg of h2b-gfp and 5 pg of bax mRNA at 1 cell stage (bax expressing cells were identified by the nuclear green signal). Cells from uninjected embryos were untreated (control group) or treated with raptinal (10 μM). At different incubation times, images were acquired with a 40 x oil-immersion objective at 28°C on a Leica TCS SP5 confocal microscope, using laser excitation of 488 nm and 633 nm. Dead cells were identified by their rounded morphology as previously reported [20], and the percentage of TO-PRO-3 positive cells analysed using ImageJ (1.53q).

### Flow cytometry

Cell viability, TO-PRO-3 uptake and ApoBD formation following apoptosis induction were determined by flow cytometry using the BD FACSCanto II flow cytometer (BD Bioscience) as previously described [33, 57]. All flow cytometry data was analysed with FlowJo software version 10.8.0 using the gating strategy described previously [33].

### Microscopy

Jurkat T cells were seeded in poly-l-lysine pre-treated 8 well Nunc® Lab-Tek® II chamber slides (Nunc, Denmark) prior to apoptosis induction and drug treatment. To detect PtdSer exposure and PANX1 channel activity, cells were stained with A5-PE and TO-PRO-3, respectively, in 1% BSA/RPMI media. For confocal microscopy analysis of ApoBDs, vesicles were seeded and stained as per Jurkat T cells. All confocal microscopy experiments were conducted at 37 °C in humidified atmosphere with 5% CO_2_ using the 63x oil immersion objective of the Zeiss Spinning Disk confocal microscope (Carl Zeiss Ltd). Image processing and analysis was performed using Zen software or ImageJ. To monitor apoptopodia formation, images were taken using a Zeiss Axio Observer Z1 microscope equipped with an Axiocam 506 monochrome digital camera and Zen software (Carl Zeiss Ltd). To quantify the extent of apoptotic cell fragmentation, image analysis was carried out in FIJI software [58] using a bespoke macro. Cell were segmented by thresholding the cytoplasm channel, from which a binary image was created. Cells that were not separated were split if necessary using a watershed. The maximum and minimum width (Feret diameters) and the perimeter was then measured for each cell.

### ATP release assay

Jurkat T cells in 1% BSA/RPMI media were seeded into a 96-well plate at 1.5 × 10^5^ cells per well and treated with anti-Fas (250 ng/mL) with or without trova (20 μM), or increasing concentrations of raptinal. After 4 h, samples were centrifuged at 3,000 x *g* for 10 mins to pellet cells and ApoBDs. The supernatant was collected and further centrifuged at 3,000 x *g* for 10 mins to remove remaining cellular debris. ATP levels in the supernatant was determined by luciferase activity assay (ATP Bioluminescence Assay Kit HS II; Roche) according to manufacturer’s instructions.

### PANX1 inhibition assay

For the long-term PANX1 inhibition assay, Jurkat T cells were induced to undergo apoptosis with increasing concentrations of raptinal in the presence of Cyto-D (10 μM). Cells treated with anti-Fas (250 ng/mL) and Cyto-D (10 μM) in the absence or presence of the PANX1 inhibitor trova (20 μM) was performed as controls. After 2 and 4 h, cells were stained with A5-FITC and TO-PRO-3 and analysed by flow cytometry (as described below). For short-term PANX1 inhibition assay, cells were first induced to undergo apoptosis with anti-Fas (250 ng/mL) in the presence of Cyto-D (10 μM). At 4 h post apoptosis induction, cells were treated with trova (20 μM) or increasing concentrations of raptinal either 10 mins prior to staining with TO-PRO-3/A5-FITC or simultaneously with TO-PRO-3/A5-FITC staining, and subsequently analysed by flow cytometry. For the PANX1 inhibitor washout assay, cells were induced to undergo apoptosis with anti-Fas (250 ng/mL) or raptinal (10 μM) with or without PANX1 inhibitors trova (20 μM) or CBX (500 μM). Cells were also treated with Cyto-D (10 μM) to limit cell fragmentation. At 4 h post apoptosis induction, cells were washed 3 x with 1% BSA/RPMI prior to staining with TO-PRO-3/A5-FITC and flow cytometry analysis.

### Immunoblotting

For the analysis of ApoBDs and Jurkat T cells, samples were lysed in lysis buffer (1% IGEPAL® CA-630, 10% glycerol, 1% Triton X-100, 150 mM NaCl, 20 mM HEPES pH 7.4, protease inhibitor cocktail tablet (Roche)) and samples were analysed by SDS-PAGE and immunoblotting using the following antibodies: rabbit anti-PANX1 (1:1000; Cell Signalling), rabbit anti-caspase 3 (1:1000; Cell Signalling), rabbit anti-ROCK1 (1:1000; Santa Cruz), mouse anti-β-actin (1:4000; Sigma-Aldrich), HRP-conjugated sheep anti-mouse Ig (1:5000; GE Healthcare), and HRP-conjugated goat anti-rabbit Ig (1:5000; GE Healthcare). For the analysis of BMDMs, precipitated supernatant was resuspended in cell extracts as previously described [28]. Proteins were separated on 15% polyacrylamide gels and transferred onto nitrocellulose membrane using Trans-Blot Turbo (Bio-Rad). Antibodies for immunoblot were anti-caspase-1 p20 (casper-1; 1:1000; Adipogen), anti-GSDMD (ab209845; Abcam; 1:1000), anti-caspase-3 (9662; 1:1000; Cell Signalling) and anti-GAPDH (ab8245; Abcam; 1:5000).

To examine effects of raptinal on the cleavage of heterologously expressed human PANX1, HEK293T cells were transiently transfected with hPANX1(TEV)-EGFP, with or without co-transfection of TEV protease, by using Polyethylenimine (PEI 25000) (Polysciences, Inc.). After ~20 h of transfection, cells were first pretreated with Cyto-D (10 μM) for 1 hr, in the presence or absence of Q-VD-OPh (10 μM), followed by raptinal (10 μM) treatment for 2 h. Cells were lysed by using PBS containing 1% Triton X-100 and a cocktail of protease inhibitors (Sigma-Aldrich). Protein samples were separated by SDS-PAGE, transferred onto 0.2 µm PVDF membrane (PALL), and blocked with 5% non-fat dry milk dissolved in a Tris-based buffer (10 mM Tris, 150 mM NaCl, and 0.1% Tween 20, pH 7.4) at room temperature for 1 hr. C-terminally EGFP-tagged human PANX1(TEV) proteins were probed with the anti-GFP antibody (Elabscience; E-AB-20086; 1:1000) or anti-Panx1 antibody (Cell Signaling; 91137 S; 1:1000). Native pro- and cleaved caspase 3 protein fragments in HEK293T cells were detected by anti-caspase 3 (T46L) antibody (Santa Cruz; sc-56055; 1:1000). Anti-β actin (AC-15) antibody (Novus Biologicals; NB600-501; 1:5000) was used as a loading control. Horseradish peroxidase (HRP)-linked secondary antibodies (Jackson ImmunoResearch, Inc.), T-Pro LumiLong Plus Chemiluminescence Detection kit (T-Pro Biotechnology), and ChemiDoc™ Imaging Systems (BioRad) were used to visualise immunoreactive signals. Uncropped immunoblots are presented in Supplemental file.

### DNA construct

Plasmid encoding hPANX1(TEV)-EGFP was generated by inserting an EGFP fragment at the 3’-end of hPANX1(TEV), an engineered human PANX1 construct with its C-terminal caspase recognition site replaced by TEV protease recognition site [30]. EGFP was first PCR amplified from pEGFP-C1 using primers 5’-CCA CGG TAC CAT GGT GAG CAA GGG C-3’/ 5’-CCA CGG TAC CCT TGT ACA GCT CGT CCA T-3’, and the amplified EGFP fragment was ligated using T4 DNA ligase at the KpnI site. Plasmid encoding TEVp was previously described [30]. All constructs were verified by using DNA sequencing.

### Whole-cell recording

Whole-cell recordings were carried out at ambient temperature using borosilicate glass patch pipettes (Harvard Apparatus) of 3 ~ 5 MΩ and an Axopatch 200 A signal amplifier (Molecular Devices). Ramp voltage clamp commands (-130 mV to +80 mV) were applied by using pCLAMP 11 software (Molecular Devices) with 7 sec intervals and an Axon Digidata 1550B (Molecular Devices) analogue-to-digital signal converter. Bath solution was composed of 140 mM NaCl, 3 mM KCl, 2 mM MgCl_2_, 2 mM CaCl_2_, 10 mM HEPES, and 10 mM glucose (pH 7.3). Internal (pipet) solution was composed of 30 mM TEACl, 100 mM CsMeSO_4_, 4 mM NaCl, 1 mM MgCl_2_, 0.5 mM CaCl_2_, 10 mM HEPES, 10 mM EGTA, 3 mM ATP-Mg, and 0.3 mM GTP-Tris (pH 7.3). Transiently transfected HEK293T cells expressing EGFP were randomly chosen for whole-cell recordings. CBX-sensitive current density was taken as the difference of currents before and after application of CBX (50 μM), at either +80 mV or -50 mV, normalised to the membrane capacitance. Bath application of raptinal (10 μM) with constant flow, followed by bath application of CBX (50 μM), was performed to examine the short-term effect of raptinal on PANX1 currents. To test the long-term effect of raptinal, transfected cells were incubated in media containing Cyto-D (10 μM), raptinal (10 μM) + Cyto-D (10 μM) for >1 h at 37 °C before recording. To examine whether endogenous caspase cascades were involved in raptinal-mediated PANX1 inhibition, cells were pre-treated with a pan-caspase inhibitor, Q-VD-OPh (10 μM), and Cyto-D (10 μM) for 1 h prior to subsequent incubation of raptinal (10 μM) for another 1 h at 37 °C before recording.

### ApoBD isolation

ApoBDs generated from Jurkat T cells treated with raptinal or UV irradiated in the presence of trova were isolated by differential centrifugation as previously described [36, 57]. Briefly, following apoptosis induction, cells were centrifuged at 300 x *g* for 10 mins to pellet cells and debris. The supernatant was further centrifuged at 3000 x *g* for 20 mins to enrich ApoBDs in the resultant pellet. ApoBD purity was determined by flow cytometry.

### Quantification and statistical analysis

No statistical methods were used to pre-determine sample size. Data are presented as the mean ± the standard error of the mean (SEM). Statistical significance was determined by either Student’s two tailed *t*-test, one-way ANOVA followed by Dunnett test or two-way ANOVA followed by Šidák test as described in the figure legend. Unless otherwise specified, at least 3 independent experiments were performed.

### Supplementary information

Supplementary Figure legend text

Supplementary Figure 1

Supplementary Figure 2

Supplementary Figure 3

Supplementary Figure 4

Supplementary Figure 5

Supplementary Figure 6

Supplementary Figure 7

Uncropped western blots

Reproducibility Checklist

## Data Availability

All datasets generated and analysed in this study are presented in this published article and its Supplementary Information files. Additional supporting data are available from the corresponding authors upon request.
