# Supplementary material for: The small molecule raptinal can simultaneously induce apoptosis and inhibit PANX1 activity
Source: Cell Death Dis. 2024 Feb 9;15(2):123. doi: 10.1038/s41419-024-06513-z (PMC10858176; doi:10.1038/s41419-024-06513-z)
Supplement: Supplementary file 1 — Supplementary Figure legend text [file 41419_2024_6513_MOESM1_ESM.docx]

**Supplementary Figure 1. Time-course analysis of raptinal induced cell death.** Flow cytometry analysis of Jurkat T cells treated with raptinal (5 μM, 10 μM, 20 μM, 40 μM) over 4 hrs to determine the relative levels of viable, apoptotic and necrotic cells. (*n* = 3) Error bars represent s.e.m.

**Supplementary Figure 2. Raptinal inhibits TO-PRO-3 uptake by apoptotic primary mouse thymocytes and zebrafish embryonic cells.** **(A)** Flow cytometry analysis of TO-PRO-3 uptake by apoptotic thymocytes treated with dexamethasone (50 μM) or raptinal (10 μM) for 4 hrs to induce apoptosis. (*n* = 3) Error bars represent s.e.m. **(B)** Confocal microscopy images monitoring TO-PRO-3 uptake by zebrafish embryonic cells overexpressing Bax to induce apoptosis, or treated with or without raptinal (10 μM). Cells that overexpress Bax also co-express H2B-GFP. **(C)** Quantification of the percentage of TO-PRO-3 positive after 1.5-2 hrs in culture under control conditions or cells overexpressing Bax or treated with raptinal, with 998, 321 and 565 cells analysed per conditions respectively in 3 independent experiments. Unpaired student’s two tailed t test was performed to determine the indicated *p* value.

**Supplementary Figure 3. Raptinal induces caspase-mediated cleavage of PANX1 during apoptosis.** **(A)** Processing of PANX1 during apoptosis was confirmed by immunoblotting. Jurkat T cells were untreated or treated with DMSO, anti-Fas (250 ng/mL), trova (20 μM, PANX1 inhibitor), anti-Fas (250 ng/mL) and trova (20 μM) or raptinal (5 or 10 μM) for 4 hrs.  **(B)** Schematic of PANX1-GFP and caspase-mediated cleavage at the C-terminus. **(C)** Expression of PANX1-GFP in Jurkat T cells was confirmed by immunoblot analysis. **(D)** Time-lapse confocal microscopy images of Jurkat T cells expressing PANX1-GFP induced to undergo apoptosis by UV irradiation (150 mJ cm^-2^). **(E)** Time-lapse confocal microscopy images of Jurkat T cells (PANX1-GFP) induced to undergo apoptosis by anti-Fas (250 ng/mL) or raptinal (10 μM) in the absence or presence of the pan-caspase inhibitor Q-VD-OPh (50 μM). Data are representative of three independent experiments.

**Supplementary Figure 4. Pan-caspase inhibitor Q-VD-OPh prohibited raptinal-induced cleavage of PANX1.** Immunoblot blots showing that raptinal-induced cleavage of caspase 3 and PANX1 were decreased by pre-incubation of HEK293T cells with a pan-caspase inhibitor, Q-VD-OPh. HEK293T cells were transfected with or without hPANX1(TEV)-EGFP or TEV protease (TEVp). Prior to protein sample collection, cells were pre-incubated with Cyto-D (10 μM) for 1 hr, with or without Q-VD-OPh (10 μM), followed by an additional 2 hrs treatment with raptinal (10 μM). Protein expressions were detected using antibodies as indicated. β-actin was used as a loading control. The p17 and p20 cleaved forms of caspase 3 represents the presence of active and partially processed forms of caspase 3, respectively.

**Supplementary Figure 5. Raptinal promotes apoptotic cell fragmentation.** Quantitation of live microscopy data from Figure 1D to determine the diameter of A5 positive cells/fragments. Reduction in the diameter of A5 positive events is indicative of more extensive apoptotic cell fragmentation. One-way ANOVA followed by Dunnett test was performed to determine the indicated *p* value.

**Supplementary Figure 6. Time-lapse microscopy analysis of apoptopodia formation during apoptosis.** Confocal microscopy images of ROCK1^-/-^ Jurkat T cells treated with or without anti-Fas (250 ng/mL), anti-Fas (250 ng/mL)and trova (20 μM) or raptinal (10 μM) over a period of 4 hrs. Data are representative of three independent experiments.

**Supplementary Figure 7. Induction of BMDM cell death by raptinal. (A)** BMDMs from WT and PANX1^nc/nc^ mice were treated with ABT-737/S6 (500 nM) or raptinal (10 μM) over 6 hrs, and LDH release was measured. **(B)** BMDMs from WT and PANX1^nc/nc^ mice were treated with the indicated concentration of ABT-737/S6 and/or raptinal for 6 hrs, and LDH release was measured. Data are representative of three independent experiments.
