# Supplementary figures and images for: The small molecule raptinal can simultaneously induce apoptosis and inhibit PANX1 activity
Source: Cell Death Dis. 2024 Feb 9;15(2):123. doi: 10.1038/s41419-024-06513-z (PMC10858176; doi:10.1038/s41419-024-06513-z)

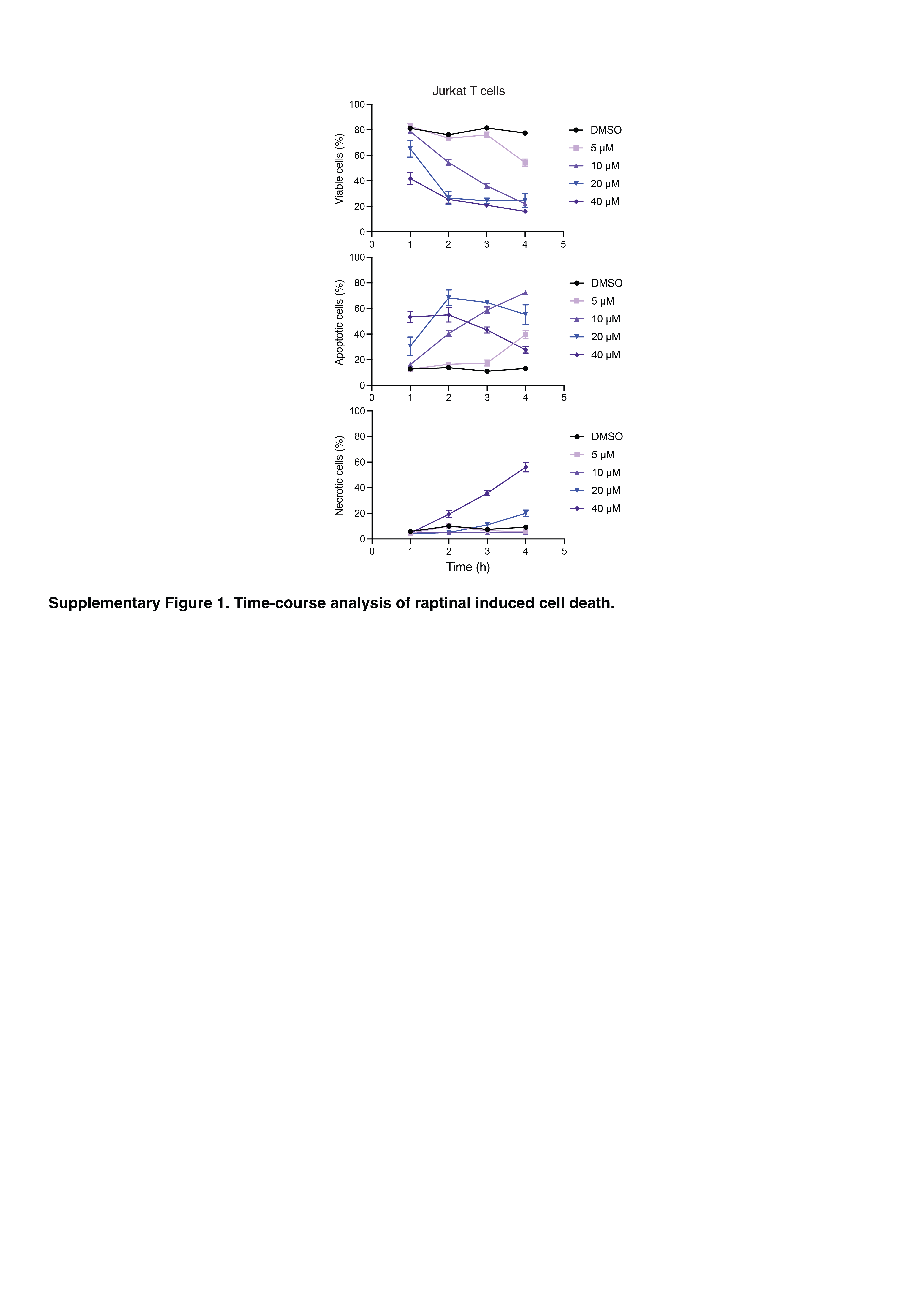

Supplement: Supplementary file 2 — Supplementary Figure 1 [file 41419_2024_6513_MOESM2_ESM.png]

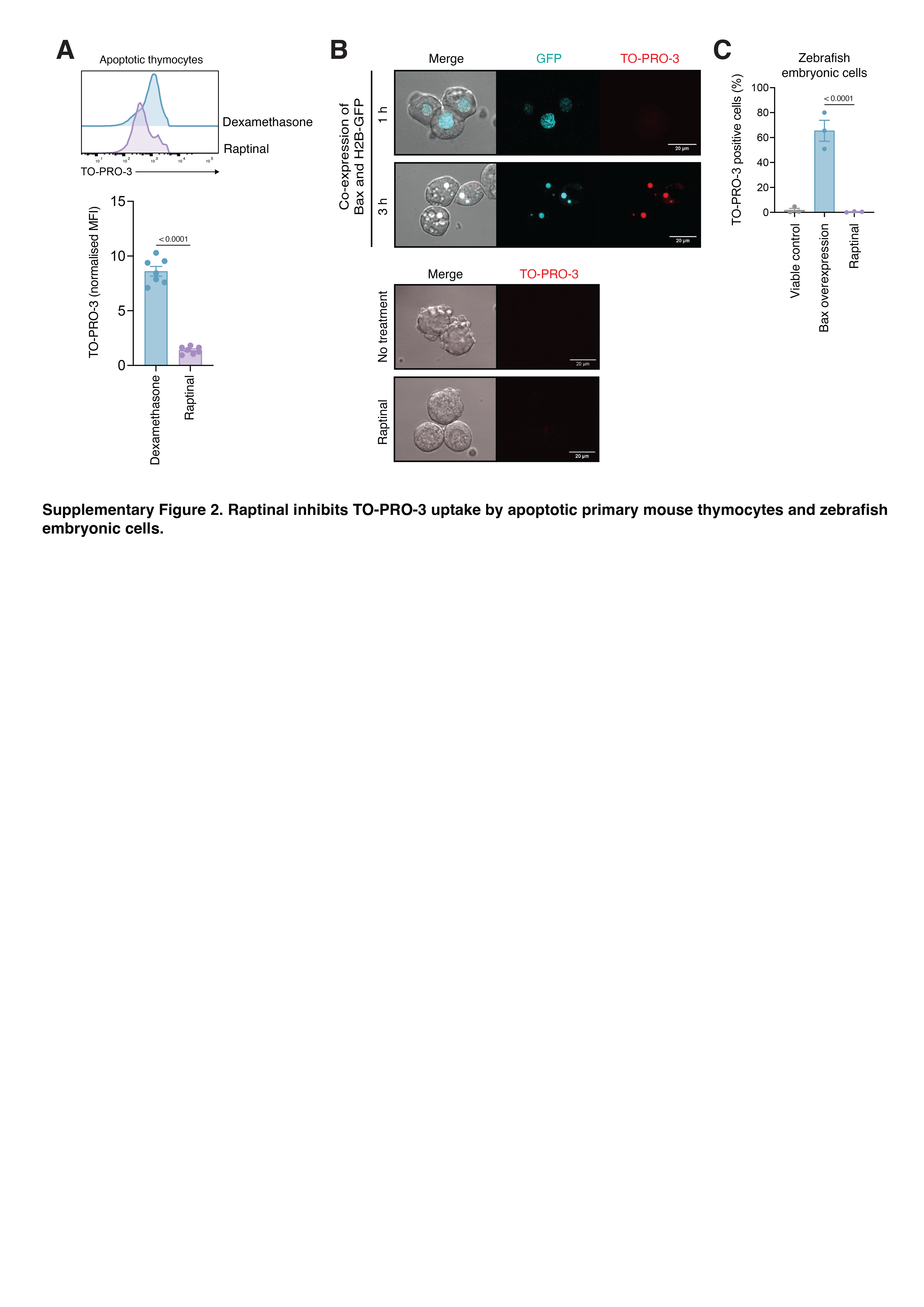

Supplement: Supplementary file 3 — Supplementary Figure 2 [file 41419_2024_6513_MOESM3_ESM.png]

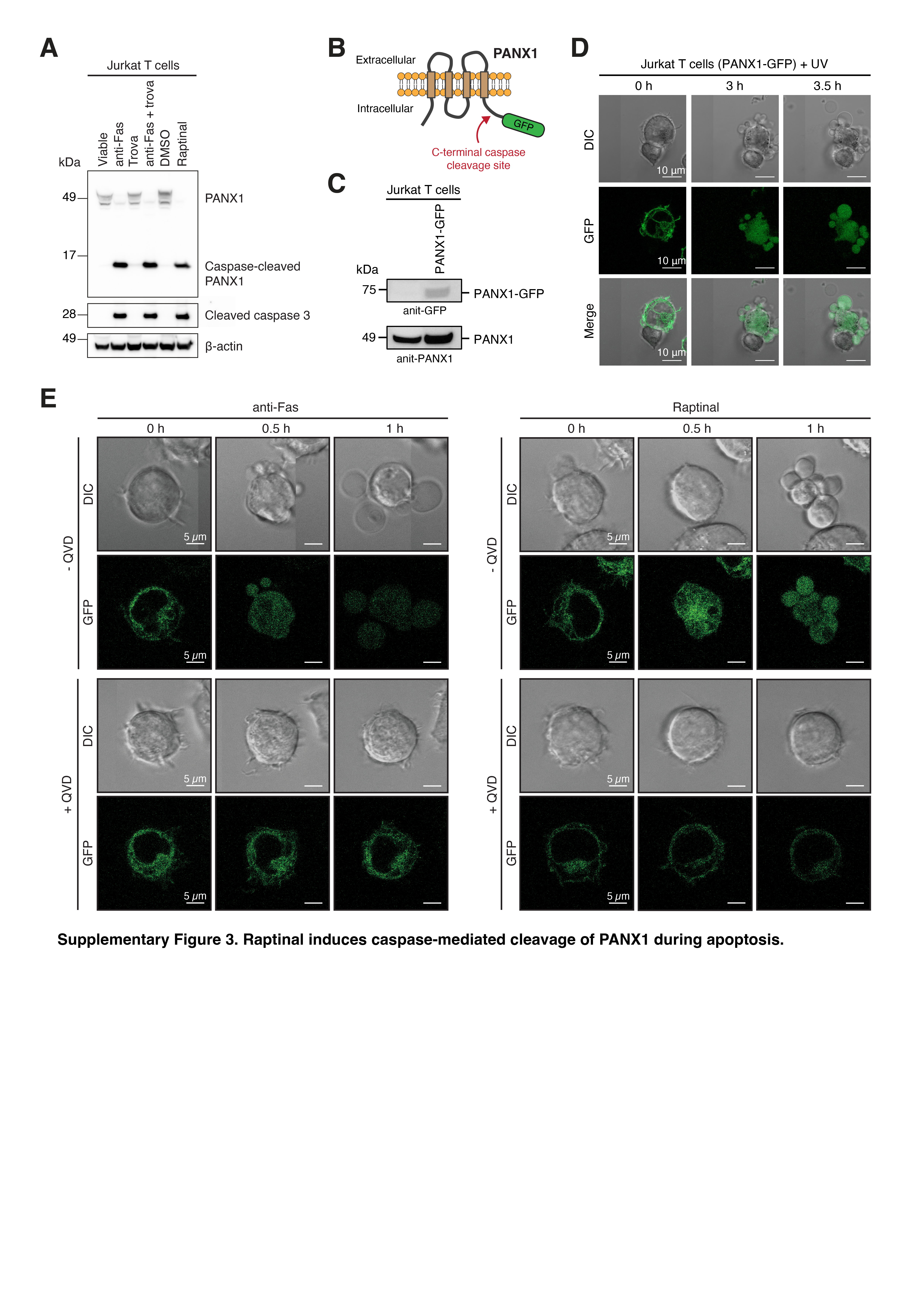

Supplement: Supplementary file 4 — Supplementary Figure 3 [file 41419_2024_6513_MOESM4_ESM.png]

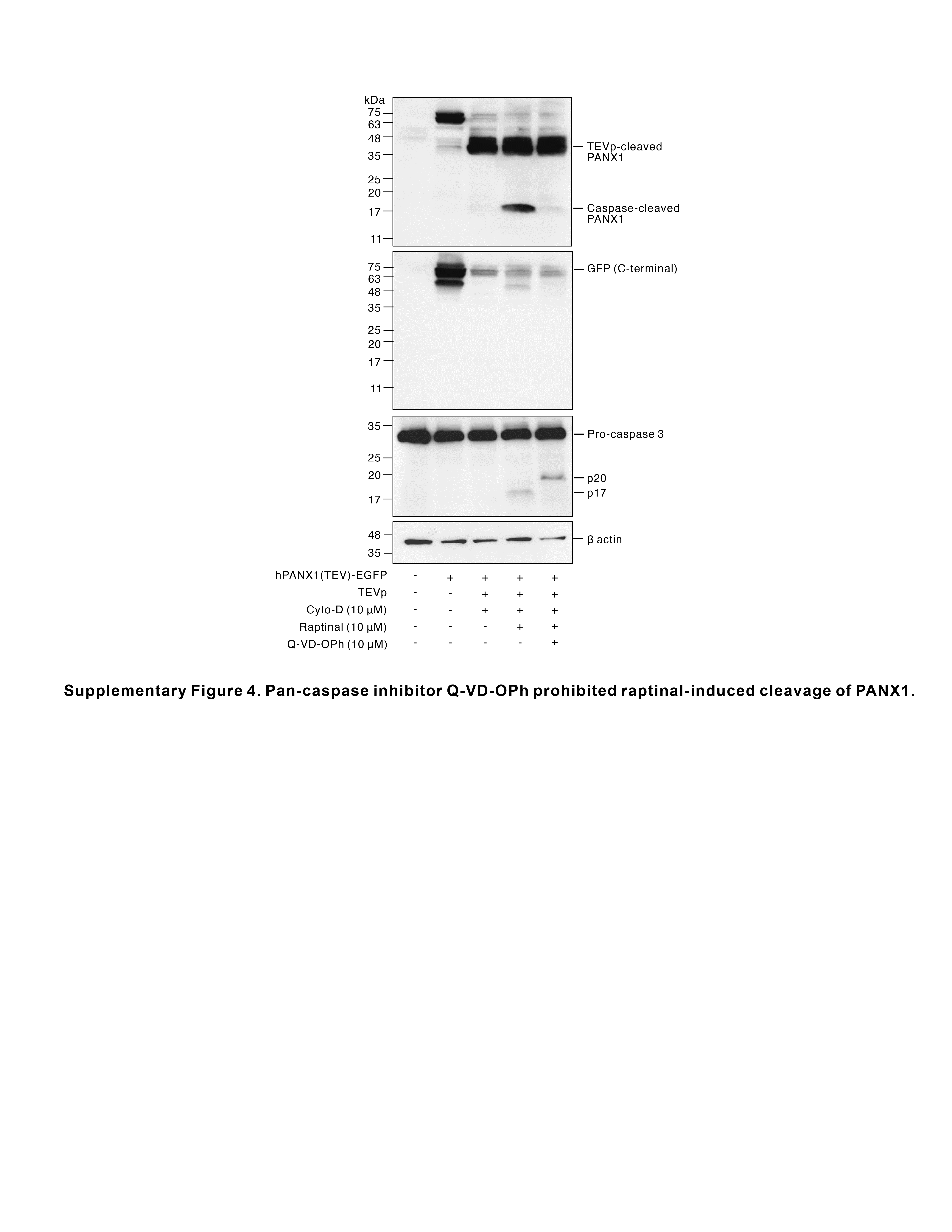

Supplement: Supplementary file 5 — Supplementary Figure 4 [file 41419_2024_6513_MOESM5_ESM.png]

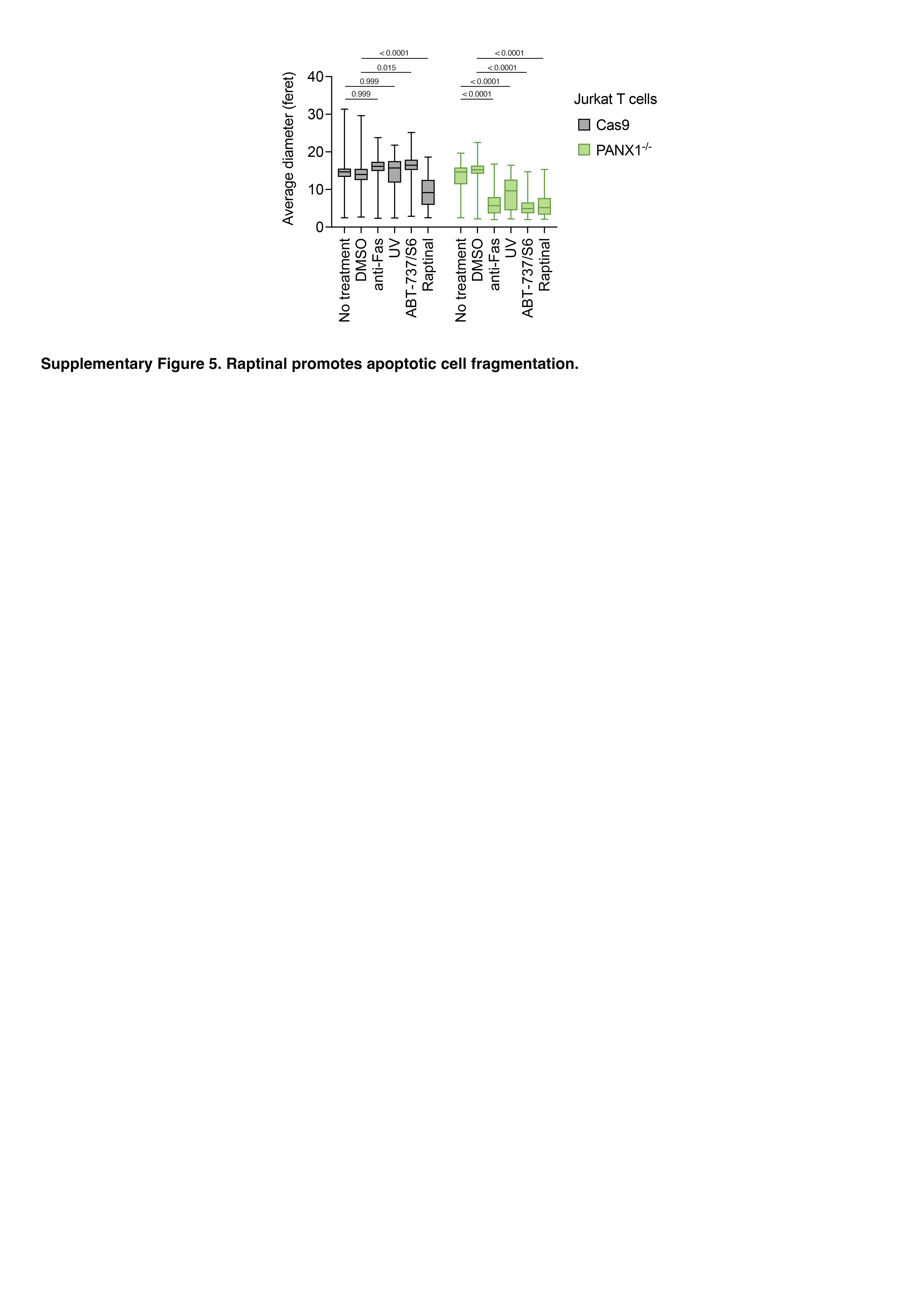

Supplement: Supplementary file 6 — Supplementary Figure 5 [file 41419_2024_6513_MOESM6_ESM.png]

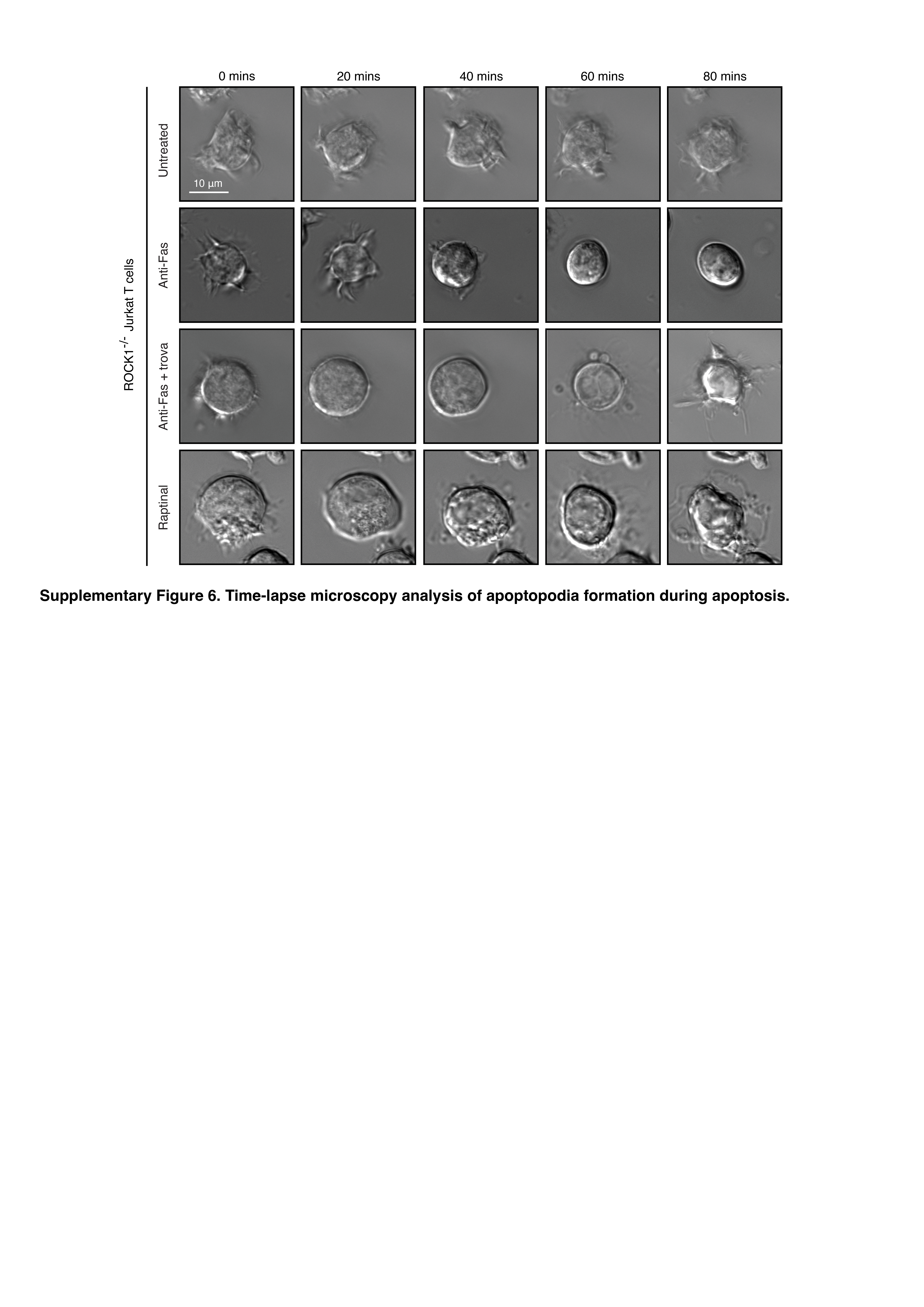

Supplement: Supplementary file 7 — Supplementary Figure 6 [file 41419_2024_6513_MOESM7_ESM.png]

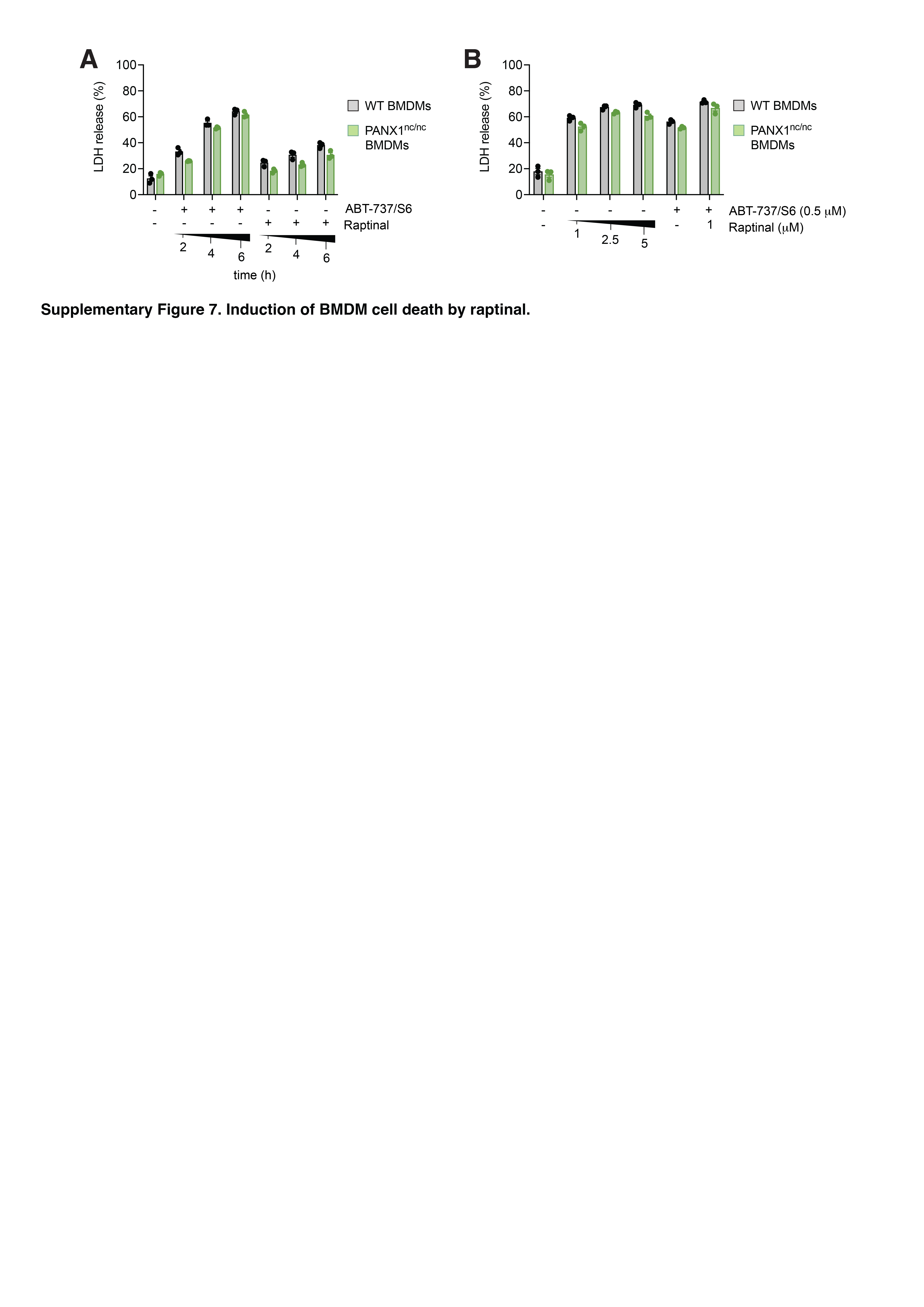

Supplement: Supplementary file 8 — Supplementary Figure 7 [file 41419_2024_6513_MOESM8_ESM.png]
