# Supplementary material for: The small molecule raptinal can simultaneously induce apoptosis and inhibit PANX1 activity
Source: Cell Death Dis. 2024 Feb 9;15(2):123. doi: 10.1038/s41419-024-06513-z (PMC10858176; doi:10.1038/s41419-024-06513-z)

Full blots for Figure 4I

α-PANX1

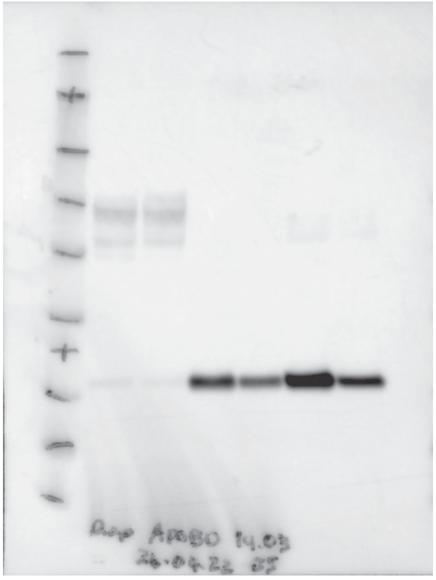

α-ROCK1

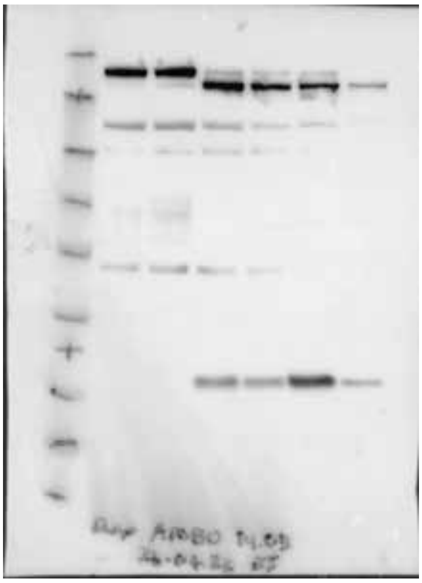

α-pro-caspase 3

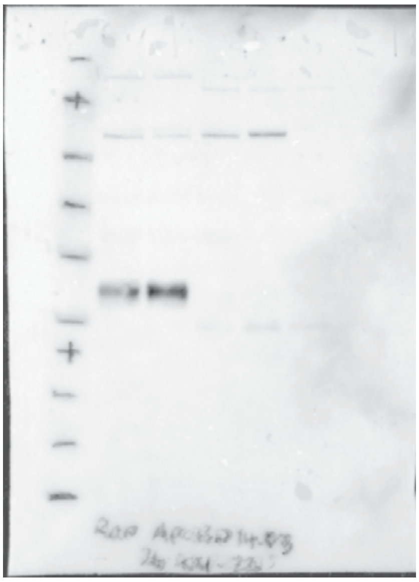

α-β-actin

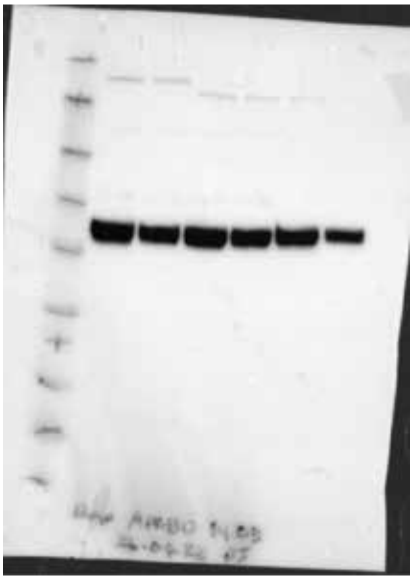

Full blots for Fig 5B

α-CASP1

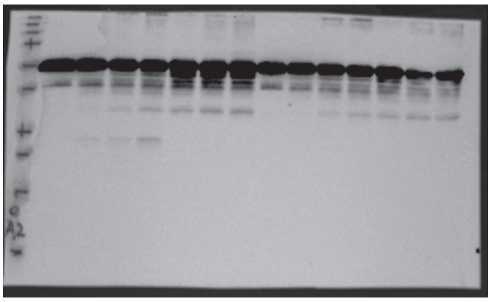

α-CASP1
Long exposure

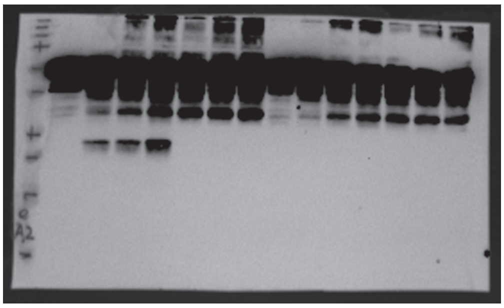

α-GSDMD

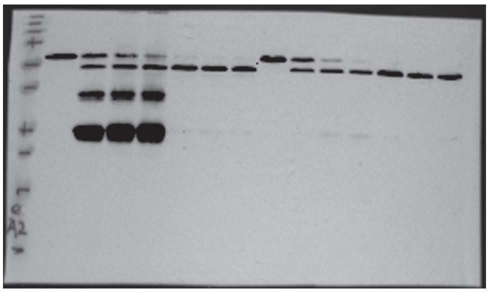

α-CASP3

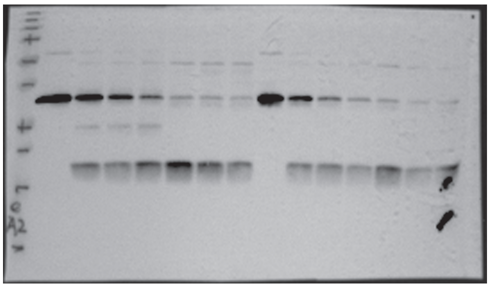

α-GAPDH

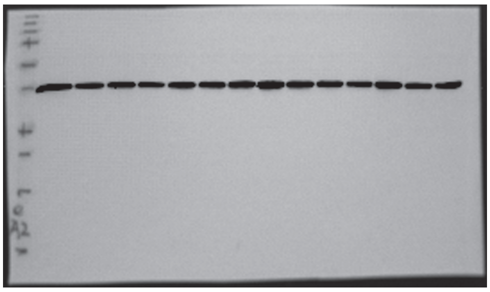

Full blots for Fig 5C

α-CASP1

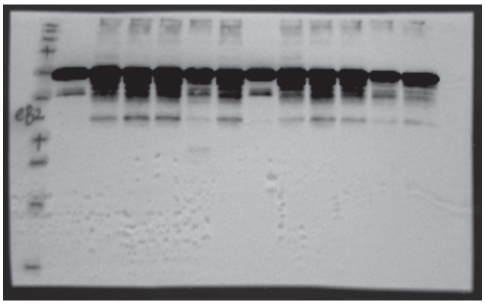

α-CASP1
Long exposure

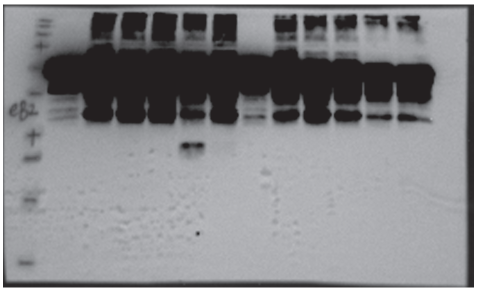

α-GSDMD

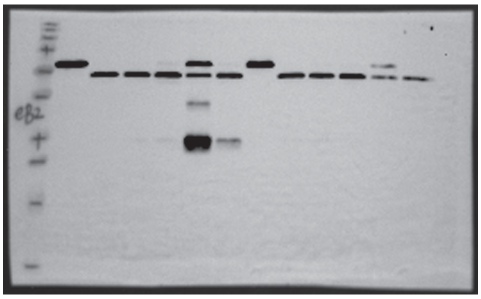

α-CASP3

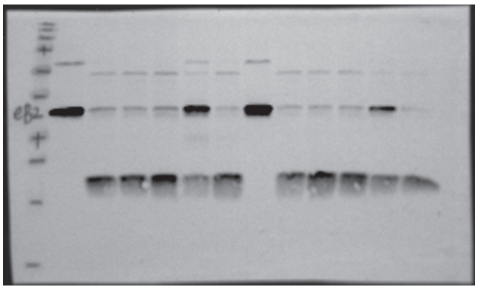

α-GAPDH

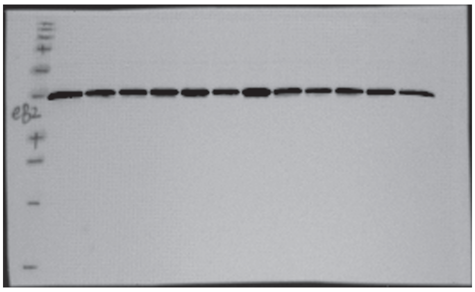

Full blots for Supplementary figure 3A

α-PANX1

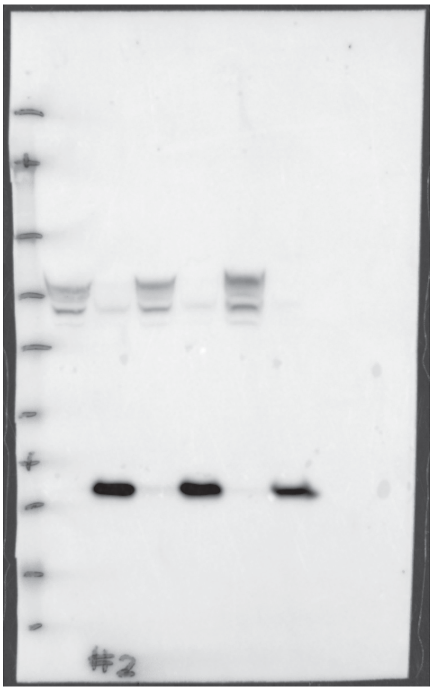

α-cleaved caspase 3

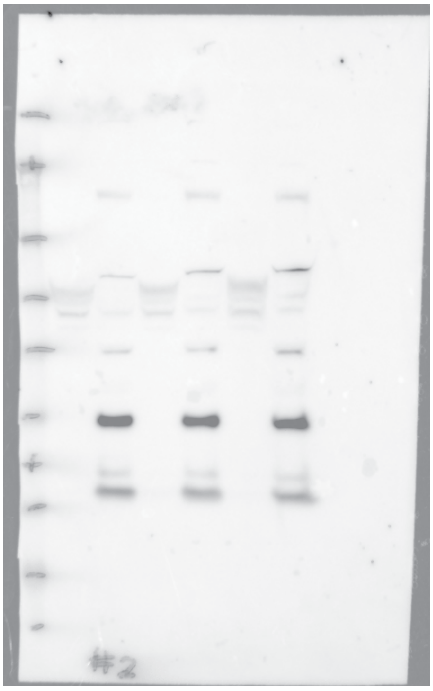

α-β-actin

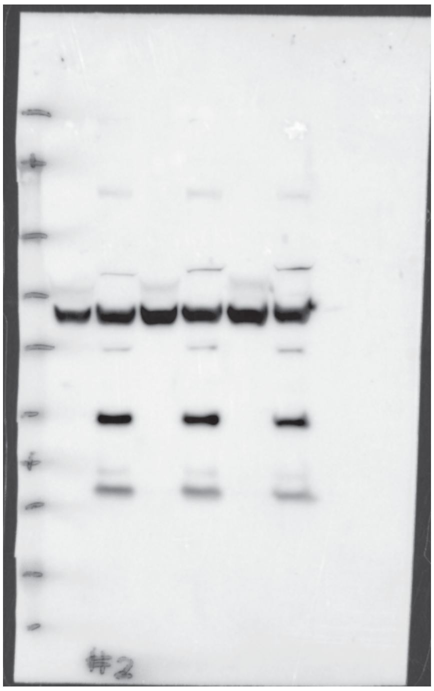

Full blots for Supplementary figure 3C

α-PANX1

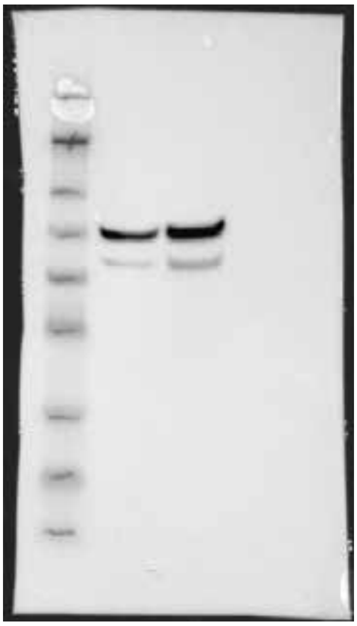

α-GFP

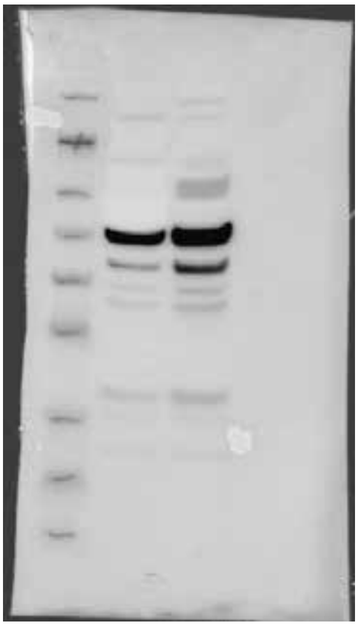

Full blots for Supplementary figure 4

α-PANX1

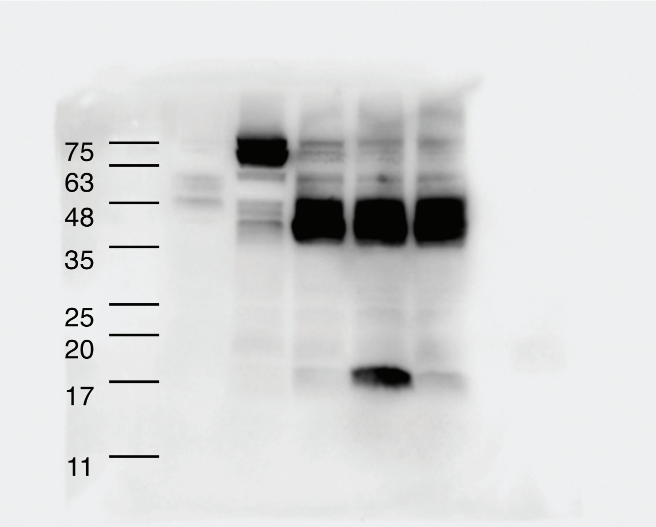

α-GFP

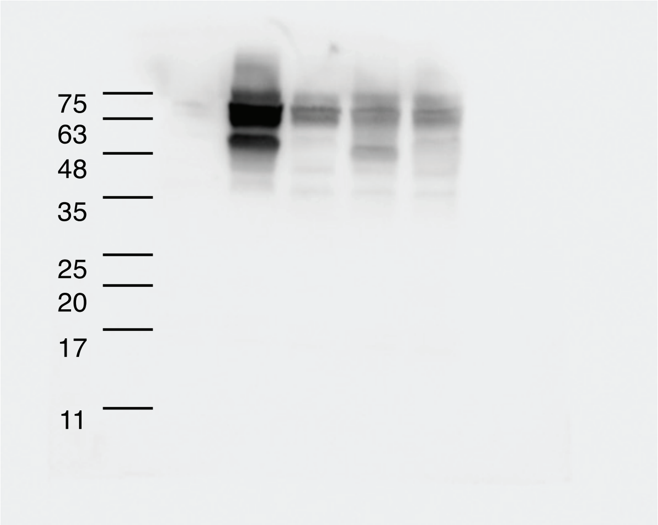

α-Caspase 3

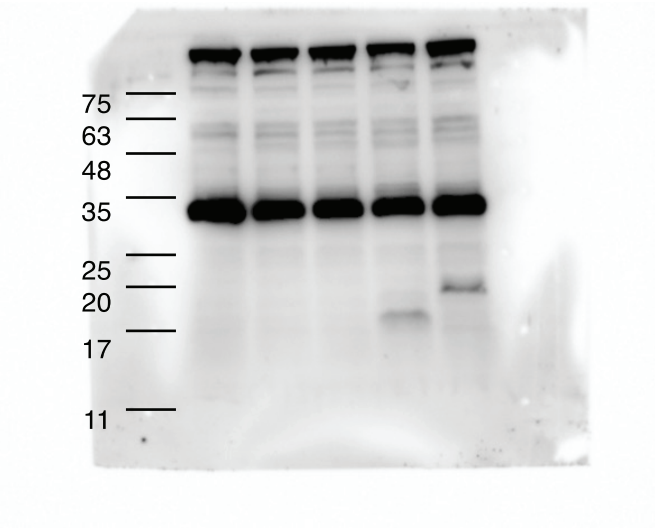

α-β actin

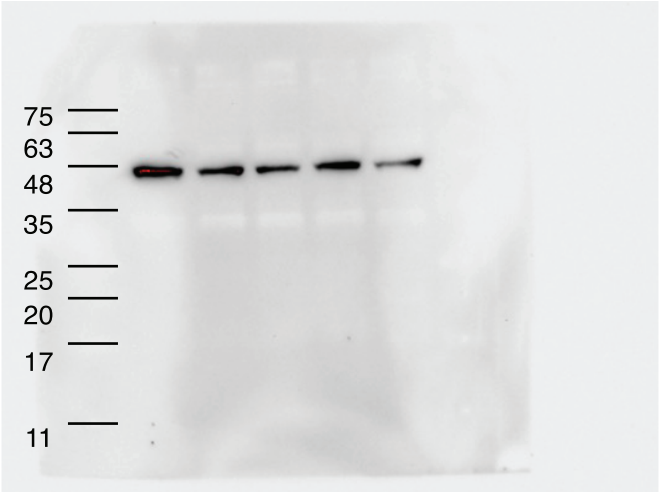

Supplement: Supplementary file 9 — Uncropped western blots [file 41419_2024_6513_MOESM9_ESM.pdf]
